# CAR-engineered cell therapies: current understandings and future perspectives

**DOI:** 10.1186/s43556-025-00401-4

**Published:** 2026-01-21

**Authors:** Mobina Bayat, Javid Sadri Nahand

**Affiliations:** 1 Molecular Medicine Research Center, Tabriz University of Medical Sciences, Tabriz, Iran; 2 Infectious and Tropical Diseases Research Center, Tabriz University of Medical Sciences, Tabriz, 15731 Iran

**Keywords:** Chimeric Antigen Receptor (CAR) Therapy, Cancers, Autoimmune Diseases, Infectious Diseases, Immunotherapy

## Abstract

Chimeric antigen receptor (CAR)–engineered cell therapies represent a significant breakthrough in immunotherapy, initially in cancer and now expanding into diverse clinical fields. While originally developed for oncology, these platforms are increasingly being adapted for non-malignant conditions such as autoimmune disorders, infectious diseases, fibrosis, ageing-related issues, and organ transplants. This review details the evolution and diversification of CAR modalities- including CAR-T, CAR-NK, CAR-macrophages, and CAR-NKT cells- as well as emerging next-generation designs. It describes the key aspects of CAR structure, signalling pathways, and manufacturing, emphasising their application in treating hematologic and solid tumours, while considering challenges such as the tumour microenvironment (TME). The review also discusses expanding uses beyond cancer- such as CD19/BCMA-targeted CAR-T cells achieving long-term remission in lupus and rheumatoid arthritis without ongoing immunosuppression, CAR-NK approaches targeting HIV, CAR-Tregs enhancing transplant tolerance, and senolytic CARs reducing tissue fibrosis. Up-to-date research through 2025 is summarised to evaluate efficacy, safety, and adverse events, noting that CAR therapies show lower cytokine release syndrome (CRS) in autoimmune diseases. Innovations like off-the-shelf allogeneic products and logic-gated CARS are highlighted, alongside ongoing challenges such as manufacturing complexity, high costs, and antigen escape. Trials like KYV-101 for multiple sclerosis demonstrate continued progress and the potential of these therapies to translate into clinical practice. Overall, CAR-engineered treatments enable precise, programmable immune modulation, paving the way for advanced therapies across an expanding array of diseases.

## Introduction

Cancer remains a significant global issue; however, recent progress in chimeric antigen receptor (CAR) cell therapies has revolutionised tumour immunology. These therapies enable the engineering of immune cells to overcome malignant immune evasion by precisely reprogramming them to recognise specific antigens without relying on major histocompatibility complex (MHC) presentation. As a result, CAR technologies have transformed cancer treatment and broadened the scope of immunotherapeutic options beyond traditional oncology [1–4]. Genetically modifying effector cells, such as T cells, natural killer (NK) cells, and macrophages, enables CAR therapies to transform antitumour immunity from passive surveillance to actively engineered responses. This method allows for targeted tumour destruction across various disease areas. However, the tumour microenvironment (TME), with its complexity and adaptability, continuously promotes tumour growth and treatment resistance [5–7]. This transition—from relying on natural immune responses to deliberately engineering immune reprogramming—has been crucial for the sustained success of CAR-T cells in treating blood cancers [2–4]. However, challenges persist, particularly in solid tumours, where the dense extracellular matrix (ECM), cellular barriers, and immunosuppressive signals from the TME impede CAR cell infiltration, persistence, and efficacy. Overcoming these obstacles calls for ongoing innovation in CAR design and a deeper understanding of TME-CAR interactions [8–12].

Beyond oncology, CAR-engineered cells are swiftly branching into treatments for non-malignant diseases, offering new options for autoimmune disorders, infectious diseases, fibrotic conditions, senescence-related diseases, and organ transplantation [13–16]. In autoimmune diseases such as systemic lupus erythematosus (SLE) and rheumatoid arthritis (RA), CAR-T cells targeting B-cell antigens such as CD19 or BCMA have achieved significant, drug-free remissions by removing autoreactive B cells and reestablishing immune tolerance. This effectiveness has been demonstrated in trials such as KYV-101 for multiple sclerosis and in vivo CD19 CAR-T for refractory SLE by 2025 [15, 17–19]. For infectious diseases, CAR-NK cells engineered to target HIV gp120 or fungal β-glucans show promise in clearing reservoirs and providing antifungal effects. Phase II trials are currently in progress for HIV [20–24]. In fibrotic diseases, fibroblast activation protein (FAP)-targeted CAR-T cells can restore tissue structure in cardiac and pulmonary models [16, 25, 26]. At the same time, uPAR/NKG2DL-directed CARs eliminate senescent cells, thereby mitigating age-related decline [14]. In transplantation, CAR-Tregs targeting HLA or OX40L help foster graft tolerance and prevent graft-versus-host disease (GVHD). Recent studies indicate they contribute to more prolonged liver graft survival [27–31]. These advancements showcase the adaptability of CAR platforms in controlling abnormal immune responses across different disease contexts.

This review offers a current and comprehensive overview of CAR cell therapy within the TME. It starts by detailing the development and mechanisms of different CAR cell types. Then, it compares the distinct features of the TMEs in solid tumours and hematological cancers, discussing how these differences influence CAR cell effectiveness. The review also critically examines key challenges, innovative engineering strategies, combination therapies, and future directions. By incorporating recent research and clinical insights, it underscores both the limitations and the promising potential of CAR-based treatments, aiming to guide future enhancements for more effective and broadly applicable cancer immunotherapies. Moreover, it considers the expanding use of CAR-engineered cells in non-malignant diseases, highlighting recent clinical advances and therapeutic possibilities.

## Types of CAR-engineered cells

**Fig. 1:**
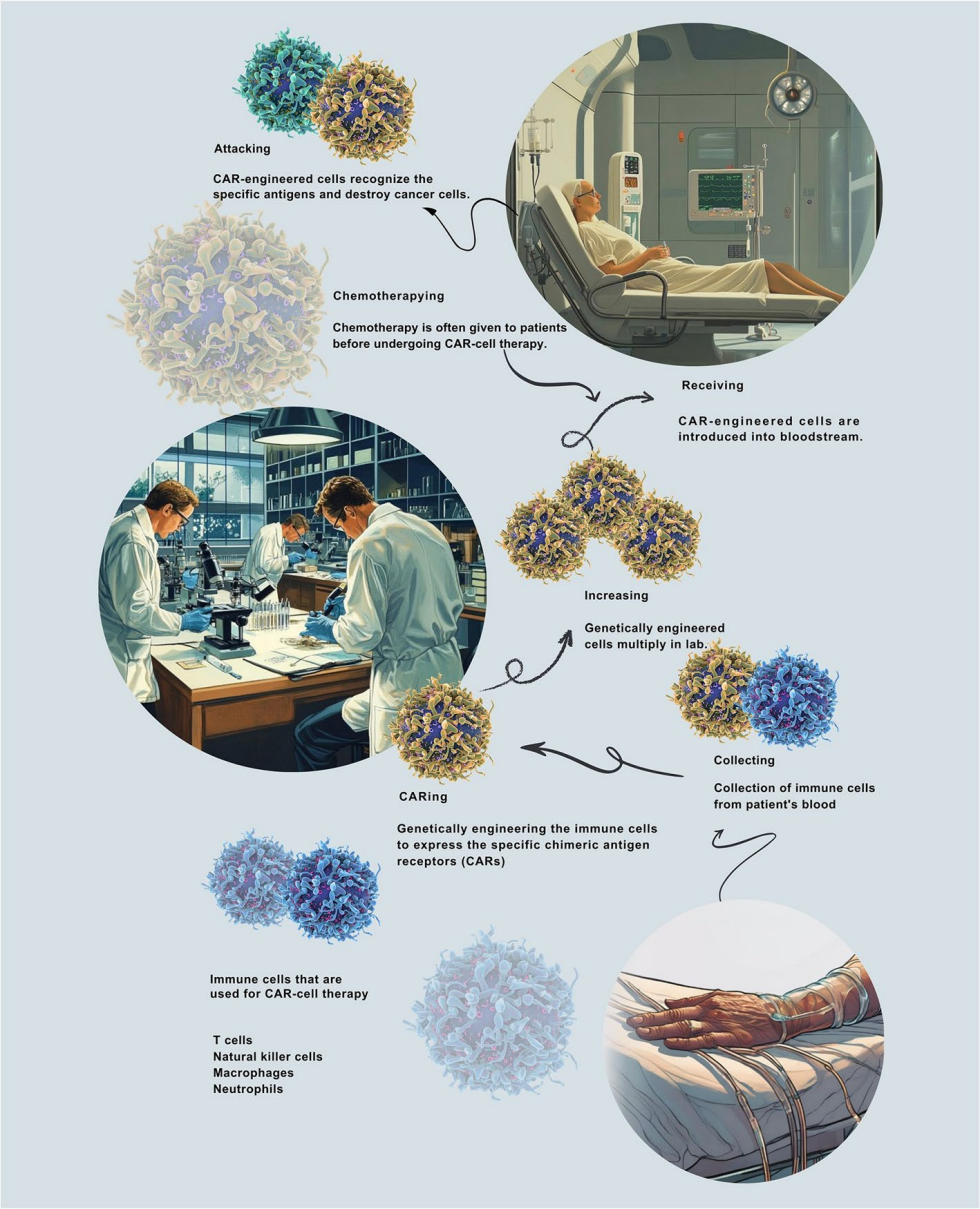
Overview of CAR-cell Generation. This diagram illustrates the complex process of creating CAR-cells. It starts with leukapheresis to collect specific immune cells from the patient's blood. These cells are then genetically modified, often using viral vectors, to express the CAR gene, which enables them to target specific cancer antigens. Next, the modified CAR cells undergo an expansion phase, during which they are grown under specific conditions to produce millions of CAR T cells. The expanded cells are then infused back into the patient, where they seek out and destroy cancer cells, providing a potent immunotherapy for certain cancers. Sometimes, patients may first receive chemotherapy to reduce cancer cell numbers and enhance the effectiveness of the CAR-cells. Abbreviation: CAR (Chimeric Antigen Receptor). This figure was created by the authors using Canva (www.canva.com)

Within the TME, tumour-associated immune cells (TAICs), such as macrophages, T cells, NK cells, and neutrophils, play roles that depend on the context, either suppressing or promoting tumour growth (Fig. 1). For example, CD8 + cytotoxic T cells target and destroy tumour cells, while CD4 + helper T cells enhance immune responses. Tumour-associated macrophages (TAMs) generally support tumour progression, whereas dendritic cells stimulate T cells via antigen presentation [11, 12]. One of the most significant advances is the development of CAR technology, which engineers’ immune cells to recognise better and destroy malignant cells. CARs are synthetic, modular receptors that direct immune cells to target tumour-specific antigens without depending on major histocompatibility complex (MHC) recognition. Introduced in 1987, CAR-T cell therapy has progressed through multiple generations, each improving therapeutic effectiveness and durability through enhanced structural elements and signalling domains [32].

Researchers are increasingly expanding CAR engineering beyond T cells to include other tumour-associated immune cells (TAICs) such as macrophages, neutrophils, B cells, and NK cells. Each cell type offers distinct advantages: CAR macrophages improve phagocytosis and antigen presentation; CAR-NK cells provide potent innate cytotoxicity with a lower risk of graft-versus-host disease; CAR-B cells produce tumour-specific antibodies; and engineered neutrophils might enhance pro-inflammatory responses within the tumour environment. This expansion of CAR platforms promotes combination strategies that utilise multiple immune mechanisms for more effective tumour targeting [33, 34].

Structurally, CARs consist of an extracellular antigen-binding domain—usually a single-chain variable fragment (scFv) from antibodies—connected via a hinge and transmembrane region to intracellular signalling modules. These often include the CD3ζ activation domain and co-stimulatory molecules like CD28 or 4-1BB, which together facilitate activation, proliferation, cytokine production, and cytotoxic effects. Different CAR generations feature various combinations of co-stimulatory and cytokine domains to enhance persistence and effectiveness. Besides conventional T cells, CAR-NK and γδ T cells combine innate and adaptive recognition, enabling effective tumour targeting even in the absence of MHC expression. CAR-M cells add mechanisms such as tumour phagocytosis and modulation of antigen presentation. In contrast, regulatory CAR-T cells (CAR-Tregs) are being developed for selective suppression of immune responses in autoimmune diseases. Expanding CAR technology across multiple immune cell types broadens the potential to overcome the complex challenges posed by the tumour microenvironment [35, 36].

CARs can identify antigens independently of MHC, enabling them to target various tumour-associated structures such as glycoproteins and glycolipids [37]. Enhancing CAR design and selecting the appropriate immune effector cells based on the tumour environment will be essential for developing therapies that are safe, effective, and personalised. The upcoming subsections explore the advantages and challenges of various CAR-engineered immune cells, highlighting their potential to elicit synergistic, long-lasting anticancer responses tailored to complex TMEs. For non-malignant applications, refer to Section “CAR-engineered cells in non-malignant diseases”.

### CAR-T cells

This subsection offers an overview of CAR-T therapy, covering T-cell roles in the tumour microenvironment, the fundamental principles and manufacturing process of CAR-T, and its significant clinical achievements in hematologic cancers. It also discusses key challenges, including toxicities, production complexity, inconsistent patient responses, and limited access. The section concludes with recent FDA approvals and new strategies aimed at enhancing effectiveness, especially in multiple myeloma and solid tumours.

T cells are a key part of TAICs and significantly influence cancer development in the TME. They include effector T cells, which perform cytotoxic functions against tumours, and exhausted T cells, which lose their activity after prolonged antigen exposure and ongoing immunosuppressive signals. The success of anti-tumour immune responses relies on the interaction between T cells and other cells in the TME. CAR-T cell therapy has revolutionised treatment for blood cancers. It is currently being investigated for solid tumours, in which a patient's own T cells are engineered to specifically target and destroy cancer cells [38, 39].

CAR-T therapy involves isolating T cells from a patient’s blood, then genetically modifying them to express CAR constructs targeting tumour-associated antigens. This targeted approach enhances tumour detection and minimises off-tumour toxicity compared to conventional chemotherapy. The manufacturing process typically takes one to six weeks and includes steps such as T-cell activation, gene transfer, and ex vivo expansion [40–44]. Patients usually undergo lymphodepleting chemotherapy before CAR-T cell therapy to promote CAR-T cell expansion, engraftment, and activity [40, 45–47]. The modified CAR-T cells are then infused in one or more doses, demonstrating notable clinical success in treating conditions such as acute lymphoblastic leukemia and various non-Hodgkin lymphoma subtypes [48–53].

**Fig. 2:**
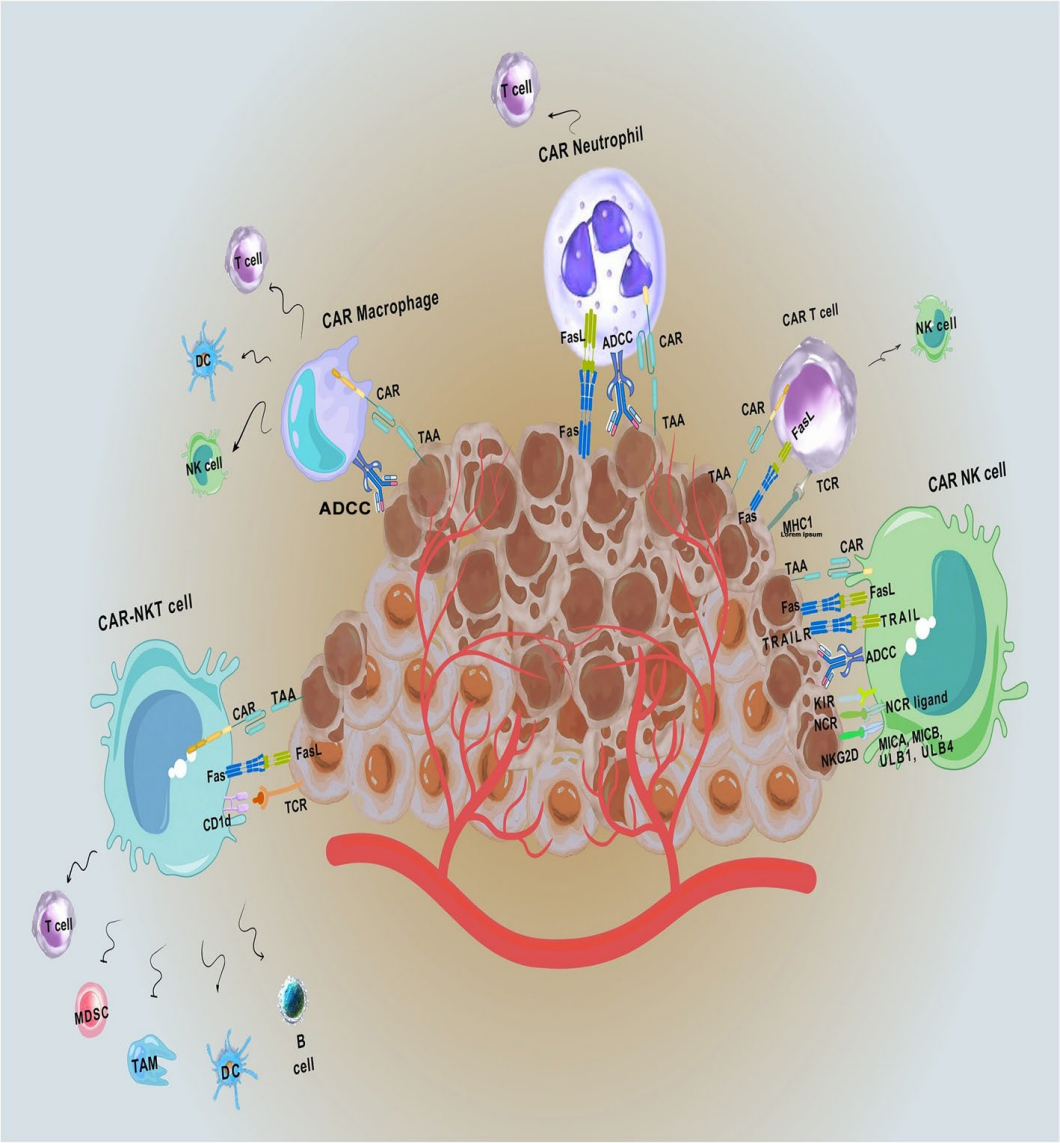
This overview describes CAR-based immunotherapies and their interactions with tumor antigens in the TME. The figure highlights the specific antigens targeted by different CAR-modified immune cells, such as CAR T-cells, CAR NK cells, CAR NKT cells, CAR-M, and CAR-N. Each CAR is engineered to recognize unique tumor-associated antigens, facilitating targeted immune responses within the TME. Arrows depict the pathways of interaction between CAR cells and other cells in the TME, illustrating how each CAR-enhanced immune cell aids in tumor recognition, immune activation, and possible tumor cell destruction. This emphasises the variety of strategies used in CAR therapy for cancer. Abbreviations: CAR (Chimeric Antigen Receptor); TME (Tumor Microenvironment); NK (Natural Killer); NKT (Natural Killer T); CAR-M (Chimeric Antigen Receptor Macrophages); CAR-N (Chimeric Antigen Receptor Neutrophils). This figure was created by the authors using Canva (www.canva.com)

Choosing the right target antigen is crucial for successful CAR-T therapy [44, 54, 55]. Ideal antigens are consistently and highly expressed on cancer cells but are not present on healthy tissues, minimising off-target effects [41, 54]. Since imperfect specificity can lead to severe toxicities, current research aims to develop strategies that more effectively differentiate tumour-specific antigens from normal ones. These include affinity tuning, combinatorial targeting, and synthetic circuits (see Fig. 2) [40, 55–58].

Although CAR-T therapy shows impressive clinical success—with response rates over 80% in some groups, especially children with acute lymphoblastic leukaemia [59–62]—these results should be viewed with caution, as several key limitations affect their broader use and application. For instance, these impressive rates should be viewed cautiously, as they mainly come from early-phase trials with highly selected patient groups with fewer comorbidities and prior treatments—factors that can artificially inflate efficacy. Selection bias, such as excluding patients with advanced disease or poor performance status, can further skew results. Additionally, prior treatments may weaken T-cell fitness, reducing CAR-T cell persistence, trafficking, and function within the tumour environment. Analysing CAR-T cell infiltration and long-term persistence is crucial to understanding their link to sustained remission, but data are limited in initial studies. The small sample sizes and single-centre design of many early trials also limit the generalisability of these findings, emphasising the need for extensive, multicentre studies to confirm these results and improve patient selection.

Beyond efficacy concerns, significant challenges persist, notably severe adverse effects such as cytokine release syndrome (CRS) and neurotoxicity [55, 56, 63–66]. CRS is characterised by a rapid release of inflammatory mediators, leading to symptoms such as fever, tachycardia, and hypotension, while neurological manifestations range from headaches to seizures [40, 55, 56, 67–69]. These side effects are usually managed with anti-IL-6 receptor antibodies and corticosteroids. Nonetheless, for enhanced safety, efforts are underway to develop genetic modifications, such as inducible suicide switches [40, 70–74].

Various CAR-T therapies have been explored for relapsed or refractory multiple myeloma (RRMM), often involving fludarabine–cyclophosphamide lymphodepletion [75–82]. Due to variations in CRS grading across studies, direct comparisons are difficult, and responses are typically evaluated using the International Myeloma Working Group (IMWG) criteria [56, 63–65]. In addition, allogeneic CAR-T therapies are under development but face challenges such as GVHD, prompting research into gene editing and iPSC-derived platforms to create universal CAR-T cells [40, 83]. Current research focuses on improving CAR-T effectiveness in solid tumours and investigating combinations with treatments such as immune checkpoint inhibitors and oncolytic viruses [84, 85].

The Drug Trials Snapshot for NIKTIMVO (axatilimab-csfr)—approved by the FDA in August 2024—summarised its use for chronic GVHD in patients ≥ 40 kg previously treated with at least two systemic therapies. Safety data from the AGAVE-201 trial (n = 79) showed that 44% of participants experienced severe adverse reactions, including infections, respiratory failure, musculoskeletal pain, and fever. Common adverse effects (≥ 15%) included elevated AST/ALT/GGT; anaemia; hypophosphatemia; fatigue; nausea; headache; diarrhoea; cough; and dyspnea. Permanent discontinuation occurred in 10% of patients, dose reductions in 8%, and dose interruptions in 44% (Table 1).

**Table 1:** Recent FDA approvals for CAR-T cell therapies

Therapy Name	Target	Disease	Phase/Approval Note	Year
Aucatzyl	CD19	Relapsed/Refractory B-cell precursor ALL	Approved (FELIX trial) Adults with r/r B-cell precursor ALL; split dosing Day 1 & 10	2024
Tecartus	CD19	Mantle Cell Lymphoma, ALL	Approved (KTE-X19 trial) Approved for r/r Mantle Cell Lymphoma and Adult ALL	2020
Kymriah	CD19	B-cell precursor ALL, DLBCL, FL	Approved (JULIET, ELARA, ELIANA, BELINDA trials)	2017
Yescarta	CD19	Large B-cell lymphoma, FL	Approved (ZUMA-1, ZUMA-5 trials)	2017
Breyanzi	CD19	Large B-cell lymphoma, FL, MCL	Approved (TRANSCEND NHL 001 trial)	2021
Abecma	BCMA	Multiple Myeloma	Approved (KarMMa trial) First FDA-approved CAR-T for multiple myeloma	2021
Carvykti	BCMA	Multiple Myeloma	Approved (CARTITUDE-1 trial)	2022
A2B530	CEA	Various cancers	Phase 1/2 (EVEREST-1 trial)	2024
TAK-102	GPC3	Hepatocellular Carcinoma (HCC)	Phase 1 Dosage: 1–2 × 10^7^ CAR-T cells/kg (after lymphodepletion)	2024
C-CAR031	GPC3	Advanced HCC	Phase 1	2024
CARv3-TEAM-E	EGFRvIII	Glioblastoma	Phase 1 (INCIPIENT trial)	2023
CART-EGFR-IL13Rα2	EGFR, IL13Rα2	Glioblastoma	Phase 1 Dosage: 1 × 10^7^ cells/kg (DL1), 2.5 × 10^7^ cells/kg (DL2)	2024
NIKTIMVO (axatilimab-csfr)	CSF-1R	Chronic GVHD (cGVHD)	Approved (AGAVE-201 trial) For cGVHD in patients ≥ 40 kg with prior treatments	2024

Data sources: U.S. Food and Drug Administration (FDA) approvals database; ClinicalTrials.gov for trial details (e.g., FELIX: NCT04404660; EVEREST-1: NCT05736731; AGAVE-201: NCT04710576)

Table 1 highlights recently approved CAR-T products, their antigenic targets, and clinical indications. While these therapies frequently achieve 70–90% response rates in hematologic malignancies, limitations persist. Trial populations are often small and highly selected, with patients possessing higher baseline T-cell fitness and fewer comorbidities. Prior therapies may impair CAR-T expansion, and long-term data on persistence remain limited. Consequently, broad real-world applicability must be interpreted carefully, emphasising the need for multicenter studies and long-term follow-up.

The risk of secondary T-cell malignancies after CAR T-cell therapy remains low, though it remains a concern [86]. Dulery et al. recently reported only a few cases out of 3,066 patients treated for hematologic cancers [86]. Additionally, the FDA's recent approval of T cell receptor (TCR) T-cell therapy marks a significant advancement in cellular immunotherapy. Unlike CAR T cells, TCR T cells recognise tumour antigens through HLA molecules. Notably, Afamitresgene autoleucel (Tecelra) received approval for unresectable or metastatic synovial cell sarcoma targeting Melanoma-associated antigen 4 (MAGE-A4), based on positive outcomes from the phase II SPEARHEAD-1 trial [87].

It should be noted that the success of CAR-T cell therapy depends on complex interactions between T-cell traits, patient-specific factors, tumour features, and the tumour microenvironment—elements that can either enhance its effectiveness or lead to failure. Important aspects include T-cell exhaustion, memory differentiation, senescence, Tregs, the balance between CD4 + and CD8 + cells, metabolic state, and TCR diversity. Exhausted T cells exhibit diminished function and correlate with poorer outcomes, whereas early memory subsets, such as TSCM and TCM, enhance persistence and antitumor activity. Senescent T cells, which are common in older patients, along with high Treg levels, tend to suppress CAR efficacy. Achieving a proper CD4 +/CD8 + ratio, supporting oxidative phosphorylation metabolism, and maintaining a diverse TCR repertoire are also crucial for improving therapy success, as they enable broader immune responses [88, 89].

Patient-related factors greatly influence CAR-T effectiveness. Higher pre-infusion levels of activated CD8 + and helper T cells are associated with better responses, whereas increased TREGs and TAMs can suppress CAR activity. Effective CAR-T expansion and long-term persistence are essential for sustained responses; inadequate expansion or early exhaustion often leads to failure. Factors like baseline T-cell health, exhaustion markers, and cytokine support affect expansion, with IL-15 receptor agonists and checkpoint inhibitors helping to improve persistence, particularly in immunosuppressive solid tumours [38, 90]. The TME also hampers CAR-T performance by inducing metabolic suppression through hypoxia and lactate accumulation. In pancreatic ductal adenocarcinoma (PDAC), hypoxia-induced HIF-1α impairs T cells, while tumour lactate from glycolysis limits T-cell proliferation and cytokine production [91, 92]. Preclinical studies show that epigenetic reprogramming with EZH2 inhibitors enhances CAR-T cell persistence and function, offering promising avenues for therapy [91, 92].

Another factor that critically influences CAR-T outcomes is tumour characteristics. Uniform antigen expression enables effective activation, whereas antigen loss or heterogeneity can lead to immune escape and relapse. A high tumour burden at infusion is associated with lower response rates due to antigen overload and immunosuppression within the TME. The design of CARS—including antigen affinity, costimulatory domains, and signalling motifs—also affects efficacy; excessive CAR density can lead to tonic signalling and cell exhaustion, whereas regulated expression improves persistence. In solid tumours, the suppressive TME limits CAR-T cell infiltration, making combination approaches targeting immune checkpoints beneficial [38]. Optimising CAR design, manufacturing processes, and patient-specific factors can improve therapy outcomes. Strategies such as emphasising early-memory T cells, reducing Tregs, and customising treatments based on T-cell biology have demonstrated improved results [88, 93]. Early identification of biomarkers, including T-cell subsets and antigen patterns, supports patient selection and treatment planning. By addressing these factors, we can achieve more durable and consistent responses in haematological cancers, advancing personalised immunotherapy tailored to each patient’s T-cell biology.

Despite these advancements, producing CAR-T cells under GMP conditions remains challenging due to scalability, reproducibility, and cost issues, mainly stemming from their autologous, patient-specific nature. The process typically takes 2–6 weeks, which can hinder treatment for patients with rapidly progressing diseases [94–97]. Additionally, donor variability complicates matters, as T cells from elderly or heavily pretreated patients often exhibit senescence, diminished proliferative capacity, and reduced cytotoxicity, resulting in inconsistent yields and increased failure rates [94, 96–98]. The intricate, multi-step process—comprising leukapheresis, viral transduction with efficiency rates of 50–70%, and extended ex vivo expansion—contributes to treatment costs often surpassing $400,000 [96, 97, 99]. Manual, open-system processes raise contamination risks and batch inconsistency, and variations in potency assays and quality-control standards across centres impede standardisation [96, 97, 100, 101].

Efforts to improve consistency focus on automation and closed-system technologies like the CliniMACS Prodigy and hollow-fibre bioreactors. While these innovations enhance sterility and process control, they remain resource-intensive and costly to implement [95, 100, 102]. Additionally, production depends on specialised personnel and extensive GMP infrastructure, restricting large-scale global deployment [96, 100]. To overcome the limitations of autologous therapies, researchers are developing 'off-the-shelf” allogeneic CAR-T cells derived from healthy donors or induced pluripotent stem cells (iPSCs). These options allow for batch production, cryopreservation, and rapid access [94, 96, 103–105]. However, they carry risks, such as GVHD and host immune rejection, which require TCR knockout using CRISPR/Cas9 or similar editing techniques. These processes could cause genomic instability and off-target effects, making regulatory approval more difficult [96, 98, 103, 105, 106]. Furthermore, the reliance of CAR-T cells on HLA reduces their universal applicability compared to CAR-NK methods [98, 103, 104, 106].

iPSC-derived CAR-T cells present a promising approach for creating standardised, expandable, and potentially universal T-cell populations. However, these products need to demonstrate safety and efficacy on par with primary T-cell–derived CARs. Achieving large-scale GMP-compliant differentiation remains technologically challenging [107–109]. Recent efforts are centred on lowering costs and streamlining production. Alternative non-viral gene delivery methods, including transposons and lipid nanoparticles, are under investigation to replace viral vectors, aiming to reduce regulatory challenges and manufacturing costs [96, 110]. Meanwhile, in vivo CAR-T generation—where CAR constructs are directly administered to patients to modify T cells—provides a groundbreaking approach that bypasses ex vivo manufacturing, accelerates timelines, and reduces costs [105, 110].

In summary, the adoption of CAR-T therapies in clinical settings remains limited due to high costs, labour-intensive autologous manufacturing, and significant logistical and regulatory challenges. These factors restrict access worldwide, especially in resource-limited regions. Autologous CAR-T cells provide personalised treatment but face issues such as variable quality, long production times, and frequent failures, making them expensive and difficult to scale. Allogeneic “off-the-shelf” options help address some of these problems but introduce risks, such as GVHD, requiring additional safety measures to edit the genes. Consequently, hybrid approaches—like in vivo CAR generation and semi-autologous iPSC-based methods—are gaining popularity as promising solutions to enhance feasibility, reduce costs, and promote more equitable access to advanced CAR therapies.

### CAR-M cells

CAR–engineered macrophages (CAR-Ms) are an emerging immunotherapy for solid tumours, offering improved tumour infiltration, enhanced phagocytosis, and the ability to modify the tumour microenvironment. This section reviews the design and development of CAR-Ms, their mechanisms of antitumor action, and the biological and clinical evidence supporting their potential. It also discusses key challenges—such as macrophage plasticity in the TME, limited ex vivo expansion, difficulties in genetic modification, and early clinical data showing limited persistence—which highlight the need for next-generation CAR-M platforms and combination approaches.

TAMs, which can make up to half of the solid tumour mass, are key drivers of tumour growth, blood vessel formation, metastasis, immune suppression, and drug resistance [7]. This makes them attractive targets for new immunotherapies. Recent biotechnology advancements now allow the engineering of macrophages with CAR constructs to improve their ability to recognise specific antigens and navigate the TME effectively [111].

CAR-engineered TAMs (CAR-Ms) are based on CAR-T technology, with an extracellular scFv domain for antigen recognition, a hinge, a transmembrane domain (often derived from CD8), and intracellular signalling domains such as CD3ζ or Fcγr. Initial approaches aimed to enhance phagocytosis; for instance, Morrissey et al. [112]. developed CAR-phagocytes (CAR-Ps), and Zhang et al. [113] designed CAR-HER2-CD147 to activate matrix metalloproteinases, breaking down the extracellular matrix to improve immune cell infiltration [111]. These developments indicate that CAR-Ms could fundamentally alter cellular interactions within the TME.

Second-generation CAR-Ms feature additional intracellular signalling domains beyond CD3ζ, which enhance antigen detection, phagocytosis, pro-inflammatory responses, and M1 polarisation. These improvements enable more sustained macrophage activation and more effective TME remodelling than first-generation CAR-Ms, although large-scale production remains challenging. Consequently, second-generation CAR-M therapies focus on improving tumour antigen recognition and sustained T-cell activation by incorporating more intracellular signalling domains. Nonetheless, challenges remain, particularly in expanding macrophages in vitro, prompting efforts to engineer bone marrow-derived macrophages for large-scale CAR production [108]. In this regard, CT-0508, an anti-HER2 CAR-M, exhibited a favourable safety profile in a Phase I trial targeting recurrent or metastatic HER2⁺ tumours (NCT04660929) and was granted FDA Fast Track status in 2021. Initial clinical results indicate manageable side effects without dose-limiting toxicities or severe CRS. At the same time, preclinical studies have shown a 60–70% reduction in tumour burden in ovarian and breast cancer PDX models through a combination of phagocytosis and TME remodelling [114]. Unlike CAR-T cells—which depend on perforin/granzyme-mediated cytotoxicity and tend to have limited effectiveness (5–20%) in solid tumours—CAR-Ms eliminate tumours via phagocytosis and release pro-inflammatory cytokines like TNF-α and IL-12, promoting infiltration of endogenous T-cells. However, CAR-Ms generally show shorter persistence, lasting days to weeks rather than months or years, as seen with CAR-T cells [38, 114].

Despite advancements, optimising cell expansion and dosing remains a challenge that requires further clinical investigation [111]. In response, Zhang et al. [115] developed CAR-engineered macrophages (CAR-iMacs) from iPSCs derived from blood monocytes, using lentiviral transduction. These achieved up to 85% CAR expression and demonstrated improved expansion and persistence. This iPSC-based strategy offers higher scalability and greater batch-to-batch consistency than autologous macrophage manufacturing [111]. Recent research indicates that silencing SIRPα in CAR-Ms markedly enhances phagocytosis and cytotoxicity against HER2-positive tumours, leading to reduced tumour growth and increased survival in mouse models. Additionally, SIRPα inhibition activates inflammatory pathways and promotes T-cell infiltration, underscoring its potential to boost CAR-M therapy in solid tumours [116].

Future third-generation CAR-Ms aim to enhance therapy by enabling in vivo reprogramming via nonviral delivery, thereby simplifying manufacturing and reducing costs. Incorporating cytokine receptor–based signalling domains is expected to improve immune regulation and tumour targeting. These advanced systems incorporate polycostimulatory signalling modules, controlled cytokine secretion circuits, and nanocarrier-based gene delivery platforms to enhance phagocytosis, boost immune cell recruitment, and more effectively modify the tumour microenvironment. In this regard, Kang et al. [117] used polymer nanocarriers to deliver CAR and IFN-γ genes, converting macrophages from tumour-supportive M2 to anti-tumour M1 phenotypes. Despite their potential, CAR-M therapies face challenges, including TAM plasticity, limited in vivo expansion, safety concerns in healthy tissues, and potential immunogenicity of CAR constructs. Clinical safety and effectiveness remain unproven, with risks such as insertional mutations from viral transfections [36]. Gaining a deeper understanding of CAR-M resistance mechanisms and the TME's immune microenvironment will be crucial for overcoming these challenges. Combining CAR-Ms with existing treatments may improve outcomes. Advances in technology are needed to enhance targeting, expansion, and safety controls [118]. The move from CAR-T to CAR-M therapies offers a transformative opportunity to use macrophages in cancer treatment. As research progresses, CAR-Ms could introduce new immunotherapy strategies to address the complexities of cancer [111].

Macrophage plasticity represents a significant biological challenge for effective CAR-M therapy. In the TME, macrophages can switch between pro-inflammatory M1 and immunosuppressive M2 phenotypes in response to cytokine signals, particularly IL-4/IL-13 and IFN-γ [114, 119–125]. This phenotypic flexibility raises the risk that CAR-Ms could revert to tumour-supportive M2 states, reducing their antitumor effectiveness and leading to therapeutic resistance. Conversely, CAR-T cells demonstrate higher phenotypic stability, although they can still become exhausted under prolonged TME stress [119, 121, 124].

To address macrophage plasticity, various engineering approaches have been designed to promote an M1-like, pro-inflammatory state. For example, CRISPR-based gene disruption of targets like SIRPα or furin improves phagocytic activity and supports continuous inflammatory signalling [119, 122]. Cytokine-armoured CAR-Ms engineered to secrete IFN-γ or resist suppressive cytokines enhance M1 polarisation and boost T-cell recruitment and local immune activation [114, 119, 123]. Additional modifications, such as the addition of TLR4 or CD3ζ–TIR signalling motifs, strengthen M1 commitment and reduce vulnerability to stimuli that induce M2 [35, 126]. Manipulating STAT6 signalling or metabolic pathways pharmacologically provides additional ways to enhance M1 stability and sustain long-lasting antitumor effects [120, 122, 127]. Together, these strategies aim to stabilise CAR-Ms in the challenging cytokine environment of the TME and prevent them from reverting to tumour-supporting phenotypes.

Manufacturing challenges further limit the development of CAR-M therapies. Macrophages have a limited ability to proliferate, usually expanding only 10–20 times outside the body, whereas CAR-T cells can expand approximately 1000-fold. As a result, GMP production demands either extensive monocyte collections or iPSC differentiation, both of which are labour-intensive and expensive and introduce variability when producing clinical doses of 10⁸–10⁹ cells [119, 121, 128–131]. Efficient gene modification remains challenging because macrophages typically show low lentiviral transduction rates (~ 30–50%), making stable CAR expression difficult. To address these issues, alternative methods such as mRNA electroporation, transient CRISPR-based systems, and in vivo reprogramming have been used to enable quick CAR expression without the need for extensive ex vivo processing [119, 124, 131–133]. iPSC-derived CAR-Ms present a scalable allogeneic platform similar to CAR-NK systems but need HLA editing or humanised CAR designs to reduce immunogenicity. Unlike CAR-NK cells, which have limited persistence, CAR-Ms show moderate in vivo longevity and carry minimal GVHD risk, making them promising options for universal off-the-shelf immunotherapy.

Early clinical evaluation of HER2-targeted CAR-M therapy, particularly CT-0508, indicates promising safety and signs of biological activity in patients with advanced HER2-positive solid tumours. In a phase I trial (NCT04660929), CT-0508 demonstrated a manageable safety profile, with no dose-limiting toxicities or severe CRS; most adverse events were mild and self-resolving. Serial tumour biopsies revealed CAR-M infiltration, activation of local myeloid cells, and increased recruitment of effector T cells, with TCR sequencing confirming the expansion of tumour-reactive clones. Approximately 44% of patients with HER2 3⁺ tumours experienced stable disease at eight weeks, while responses in HER2 2⁺ tumours were limited. Despite these encouraging mechanistic insights, the small, diverse, and heavily pretreated cohort restricts definitive conclusions. Furthermore, the transient persistence of CAR-M cells highlights the necessity for improved durability. These results support ongoing optimisation and rational combination approaches, such as pairing CAR-Ms with checkpoint inhibitors to enhance therapeutic efficacy [134–137].

Although CAR-M therapy shows increasing clinical potential, it remains limited by significant manufacturing challenges, high costs, and logistical hurdles similar to those encountered by CAR-T platforms. The limited ability of macrophages to proliferate means that resource-heavy monocyte collection or iPSC differentiation is necessary, and centralised manufacturing further restricts patient access [138, 139]. Emerging in vivo reprogramming methods, like nanoparticle-based CAR delivery, could lower costs and streamline manufacturing. However, they need thorough clinical testing to ensure safety, scalability, and effectiveness in various patient groups [117, 138].

### CAR-NK cells

CAR-NK cell therapy has become a rapidly progressing and highly anticipated approach in cancer immunotherapy due to its favourable safety profile, natural antitumor capabilities, and compatibility with allogeneic “off-the-shelf” production. This section reviews the biological traits that set NK cells apart from T cells, describes the design and mechanisms of action of CAR-NK platforms, and assesses the preclinical and clinical data supporting their therapeutic promise. It also highlights key challenges hindering CAR-NK effectiveness—such as limited in vivo persistence, telomere-driven ageing, donor variability, and manufacturing hurdles—offering an overview of the opportunities and obstacles to advancing CAR-NK treatments.

CAR-NK therapy is generally viewed as safer and potentially more effective than CAR-T therapy in many cases, mainly because NK cell activation causes fewer inflammatory toxicities. Unlike CAR-T cells, which often cause CRS and neurotoxicity, NK cells have a higher activation threshold and release cytokines more controllably, resulting in gentler immune responses [140]. Early clinical data show that patients treated with CAR-NK experience minimal neurotoxicity, likely due to lower cytokine levels and reduced NK cell proliferation, both of which help prevent immune cells from infiltrating the central nervous system [141].

The superior safety profile of CAR-NK cells is supported by both mechanistic and clinical evidence. Since NK cells lack TCRs, they do not initiate alloreactive responses and therefore do not cause GVHD in allogeneic or HLA-mismatched settings [142–146]. This distinctive characteristic enables scalable, off-the-shelf CAR-NK products without the need for TCR knockout, which is required for allogeneic CAR-T manufacturing. Moreover, CAR-NK cells produce lower levels of CRS-related cytokines, such as IL-6 and TNF-α, while mainly secreting IFN-γ and GM-CSF, cytokines associated with less systemic toxicity [143, 144, 146–148].

CAR-NK cells maintain antitumor activity via both CAR-dependent and innate NK mechanisms, including ADCC, perforin/granzyme release, and recognition of stress ligands, enabling them to kill even antigen-loss variants. Their relatively short lifespan in the body (days to weeks) acts as a natural safety switch, reducing long-term toxicities, unlike CAR-T cells that can persist for months or years. Clinical trials with CAR-NK cells consistently report low severe CRS rates (0–7%, compared to 10–30% with CAR-T) and almost no neurotoxicity, with no cases of GVHD even in mismatched allogeneic settings [143–151]. Researchers are exploring engineering methods, such as membrane-bound IL-15, to boost persistence without compromising safety.

In addition to improving safety, CAR-NK cells address significant limitations of CAR-T cells, such as the need for prior antigen exposure and the risk of GVHD. NK cells naturally detect malignant or stressed cells via germline-encoded receptors. They can be produced from umbilical cord blood, peripheral blood, cell lines, or iPSCs, allowing for scalable allogeneic manufacturing [152]. While CAR-T treatments usually cost over $400,000–$450,000 per patient in the US and Europe, the complete pricing for CAR-NK therapies remains unclear due to limited commercialisation. However, off-the-shelf NK products are expected to be much more affordable [141, 153].

Most CAR-NK studies have primarily focused on hematologic malignancies. The iPSC-derived CD19 CAR-NK product FT596 demonstrated significant efficacy, with responses observed in 5 of 8 patients as monotherapy and 4 of 9 when combined with anti-CD20 antibodies [154]. In a Phase I trial, a single dose of ≥ 90 million CAR-NK cells resulted in objective responses in 73% (8/11) of patients, including multiple complete remissions, without any notable toxicity [150]. Cord blood-derived CD19 CAR-NK cells showed responses in 48.6% of patients with B-cell malignancies [151]. Additionally, early clinical trials of HER2-targeted CAR-NK cells in neuroblastoma reported promising safety profiles and initial signs of antitumor activity [155]. In AML, CAR-NK cells targeting CD123 or CD33 have shown strong cytotoxic effects in preclinical studies, but limited persistence has restricted their clinical impact. Adding IL-15 transgenes has enhanced NK cell survival and cytotoxicity, though it has sometimes increased toxicity in animal models. Current trials are testing CAR-NK cells combined with chemotherapy and investigating new NK activators, such as NKG2D-based CARs and dual-target strategies targeting CD33/CLL1 [152].

Despite encouraging clinical results, significant hurdles remain before CAR-NK therapies become widely available. Safety profiles depend on factors such as NK cell source, expansion techniques, and individual patient characteristics, including disease burden. Although activated NK cells produce fewer inflammatory cytokines than T cells, they can still trigger myeloid cell activation. Ongoing research is exploring genetic methods to reduce NK cell inflammatory mediators, aiming to enhance safety [156].

Technological constraints also influence the development of CAR-NK cells. NK-92 cells, although approved by the FDA for clinical applications, lack CD16, need irradiation, and thus cannot survive long in vivo. Primary NK cells require ex vivo expansion, which adds to manufacturing complexity and cost. Additionally, excessive cytokine priming can lead to 'cytokine addiction,' reducing their persistence in vivo. Cryopreservation further affects cell viability and functional recovery, highlighting the need for optimised freeze–thaw protocols [154].

Biological limitations in NK cells include telomere shortening and replicative senescence. Unlike T cells, NK cells have limited telomerase activity and experience telomere loss during expansion, which reduces their cytotoxicity and shortens their lifespan (days to weeks, sometimes longer with IL-15 support) [144, 150, 157–159]. While this brief window improves safety, it limits long-term responses. To address this, strategies like IL-15 or Neo-2/15 support, TERT overexpression to lengthen telomeres, and co-stimulatory domains such as 4-1BB or OX40 are being investigated to enhance persistence without compromising safety [144, 150, 157–159].

In addition, at the manufacturing stage, challenges such as donor variability, limited expansion (10–100-fold versus approximately 1000-fold for CAR-T), and viability loss during cryopreservation remain significant obstacles. iPSC-derived NK platforms overcome these issues by allowing standardized, unlimited production with high gene-editing efficiency (20–70% transduction) and suitability for bioreactor-based scaling [144, 160, 161]. For off-the-shelf applications, CAR-NK cells are advantageous because they do not cause GVHD, face fewer HLA-related limitations, and offer more reliable expansion and manufacturing prospects than CAR-M and CAR-neutrophil products [144, 160].

Although CAR-NK therapies are anticipated to be more cost-effective than CAR-T treatments, they still face challenges in large-scale production, distribution logistics, and regulatory compliance. Manufacturing costs are currently high, ranging from $100,000 to $200,000 per dose. Regulatory frameworks need to ensure genetic stability, safety of allogeneic products, and consistent potency across different manufacturing batches [148, 161–164]. Even with advances toward universal iPSC-derived platforms, global accessibility remains limited due to the small number of specialised cell-therapy centres.

### CAR-NKT cells

CAR-modified natural killer T (NKT) cells, known as CAR-NKT cells, represent a promising new approach in cancer immunotherapy. They combine features of T cells and NK cells, providing a hybrid effector system with rapid cytokine responses and innate-like tumour recognition [165]. This subsection reviews the biological and immunological features that distinguish CAR-NKT cells from CAR-T and CAR-NK therapies, highlights key preclinical and early clinical findings, and explores the potential and challenges of using CAR-NKT cells as both autologous and allogeneic therapies.

The manufacturing process for CAR-NKT cells shares similarities with those for CAR-T and CAR-NK cells, but is more complex due to the low abundance of NKT cells in peripheral blood. Initially, NKT cells are isolated from patient or donor samples, then activated and expanded outside the body. Afterwards, CAR constructs are introduced—typically using viral transduction—and the modified cells are infused back into the patient. This method boosts NKT cells' ability to target tumour-associated antigens (TAAs), infiltrate tumours, and perform cytotoxic and immunomodulatory roles within the TME [37].

A key benefit of CAR-NKT cells is their distinct antigen-recognition mechanism. Unlike conventional αβ T cells, which identify peptide antigens via diverse MHC molecules, NKT cells are limited by monomorphic CD1d and recognise lipid antigens [166, 167]. This restriction allows CAR-NKT cells to be engineered with CARs targeting TAAs and to harness their natural ability to detect lipids presented by CD1d, broadening their effectiveness against tumours that escape standard MHC-based immunity. Furthermore, their quick cytokine release and ability to infiltrate tumour sites help enhance modulation of the TME [165–167].

Safety is a key advantage of CAR-NKT platforms. While CAR-T therapies are effective clinically, they often cause CRS, neurotoxicity, and GVHD in allogeneic settings. Conversely, initial clinical data on CAR-NKT cells show a favourable safety profile, with little to no CRS or neurotoxicity and a reduced risk of GVHD [168, 169]. For instance, a pediatric neuroblastoma trial using CAR-NKT cells reported no CRS or neurotoxicity, likely due to their low IL-6 production and limited in vivo expansion [166]. The lower GVHD risk is mainly due to the invariant Vα24-Jα18 TCR, which recognises the monomorphic CD1d rather than the polymorphic HLA, thereby reducing the alloreactivity observed in traditional T cells [169]—these features position CAR-NKT cells as promising candidates for “off-the-shelf” allogeneic treatments.

Clinical data indicate that allogeneic CAR-NKT approaches are feasible. In the Phase I ANCHOR trial (NCT00840853), Ramos and colleagues infused five patients with relapsed or refractory B-cell NHL or ALL with HLA-unmatched CAR-NKT cells that co-expressed a CD19 CAR, IL-15, and shRNAs targeting HLA class I and II [170]. No GVHD was observed, even at higher doses, and safety was managed effectively, with minimal CRS and no neurotoxicity. Responses included partial remissions in NHL and a complete remission in ALL. However, these encouraging findings should be interpreted with caution. Factors such as residual contamination with conventional T cells (as low as 0.04%), patient immune status, the extent of HLA mismatch, and the inflammatory cytokine environment could still lead to alloreactivity [170]. Moreover, while reducing HLA molecules decreases host T-cell recognition, it may theoretically raise the risk of NK-cell–mediated rejection in mismatched recipients. However, this was not seen in the trial. Ongoing gene-editing strategies focus on eliminating residual TCR expression and refining HLA modulation to reduce these risks further [170, 171].

Much progress has been made in creating universal CAR-NKT (UCAR-NKT) platforms. Li and colleagues engineered UCAR-NKT cells by combining invariant NKT TCRs with HLA gene editing in hematopoietic stem cells (HSCs), producing allogeneic cells with strong antitumor effects against both blood cancers and solid tumours [171]. These UCAR-NKT cells also modify the TME by reducing immunosuppressive TAMs and myeloid-derived suppressor cells (MDSCs), thus boosting the body's own immune response [171]. Preclinical studies show they are safe, with low risks of GVHD and CRS, supporting their potential as a scalable universal cell therapy [171]. Evidence from preclinical models shows a synergy between allogeneic CAR-NKT cells and autologous CD8⁺ T cells, indicating that combining these approaches could better control tumours [172, 173]. Economically, CAR-NKT therapies may be more cost-effective than CAR-T therapies in the long run due to simpler allogeneic manufacturing and lower costs for managing severe side effects [165].

Preclinical studies highlight the advantages of CAR-NKT cells over traditional CAR-T cells in specific scenarios. Rotolo et al. found that CD1d-restricted CD19-CAR-NKT cells outperformed CD19-CAR-T cells in targeting CD1d-positive lymphomas, achieving significant tumour reduction after a single dose without causing GVHD [174]. Another study showed that α-GalCer–pulsed CAR-NKT cells had potent built-in cytotoxicity through perforin release and effectively targeted CD1d-expressing human T-cell leukaemia lines, unlike conventional CD8 + T cells, which did not respond as effectively [169]. In addition to direct tumour destruction, CAR-NKT cells enhance CD8 + T-cell cross-priming and support more sustained tumour control in vivo [175]. Overall, these results demonstrate that CAR-NKT cells are versatile effectors, offering both direct cell killing and immune regulation within the TME.

Despite their promising features, CAR-NKT therapies encounter significant hurdles. The low abundance of NKT cells in peripheral blood limits the available starting material. It makes ex vivo expansion more difficult, leading to more protracted, more costly, and more variable manufacturing processes [176, 177]. Differences among NKT subsets and immunosuppressive signals in the TME can also hinder their persistence and effectiveness in vivo [170, 176, 177]. Regulatory and translational considerations, including maintaining product stability, consistent potency, and avoiding GVHD, are crucial, especially as more complex gene edits are implemented [177, 178]. Although autologous methods are logistically challenging and limit accessibility, recent progress in allogeneic HSC-engineered CAR-NKT platforms shows promise for higher yield, purity, and scalability, which could lower costs and streamline regulatory approval [171, 177, 178]. Still, more clinical validation and long-term follow-up are essential to demonstrate sustained efficacy and safety across diverse patient groups.

Outside of oncology, CAR-NKT cells are also being explored for non-cancer conditions. Their ability to regulate immune responses makes them promising for autoimmune diseases, where they could reduce harmful immune activity without causing widespread immunosuppression. They may also be helpful in infectious diseases by providing quick, cytokine-mediated antiviral effects [165]. Overall, CAR-NKT cells offer a new way to combine innate and adaptive immunity, with a growing safety record in clinical use, especially in allogeneic settings. To unlock their full potential against cancer and other diseases, ongoing improvements in CAR design, antigen targeting, and overcoming TME barriers are crucial [165, 170–178].

### CAR-Neutrophils

Neutrophils, the main circulating leukocytes in humans, are frequently found in various tumour types and can constitute a significant proportion of tumour-infiltrating immune cells [179–182]. Their significant heterogeneity and functional flexibility in the TME allow them to play both pro-tumour and anti-tumour roles at different stages of tumour development [183]. Tumour-associated neutrophils can directly kill solid tumours and are involved in antibody-dependent cell-mediated cytotoxicity (ADCC) [184, 185]. However, they can also promote angiogenesis, facilitate metastasis, and suppress the anti-tumour activity of other immune cells within the TME [186, 187]. Neutrophil subsets that support tumours can reduce the effectiveness of standard treatments, including immunotherapies, driving interest in strategies targeting neutrophils across various cancers [188]. Nonetheless, because neutrophils in the TME are highly diverse [186, 187], broad suppression with small molecules or antibodies risks eliminating both beneficial and harmful neutrophil subsets, potentially causing neutropenia and raising infection risks in cancer patients [183, 189]. These challenges highlight the importance of developing more targeted approaches—such as chimeric antigen receptor–modified neutrophils (CAR-neutrophils, CAR-N)—to leverage their anti-tumour effects while limiting collateral damage [183].

A notable study by Chang et al. [183, 190]. demonstrated the effective use of CAR technology in neutrophils. They engineered CAR-neutrophils with a chlorotoxin (CLTX)–based CAR (CLTX-T-CAR) that targets glioblastoma (GBM) markers and signals explicitly through CD3ζ. To address the challenge of directly engineering mature neutrophils, the CLTX-T-CAR was inserted into a safe-harbour locus in human embryonic stem cells (hESCs), which were then differentiated into neutrophils. These hESC-derived CAR-neutrophils rapidly formed immunological synapses with target cells and exhibited increased phagocytosis, reactive oxygen species (ROS) production, and neutrophil extracellular trap (NET) formation. Significantly, unlike primary neutrophils, CAR-N cells maintained an N1-like antitumor phenotype even in an immunosuppressive TME both in vitro and in vivo. Furthermore, CAR-neutrophils were loaded with nanodrugs such as silica nanoparticles containing chemotherapeutic or radiation agents, which were released upon target engagement, working synergistically to inhibit tumour growth in GBM mouse models [183, 190, 191]. This study demonstrates how combining CAR engineering with nanotechnology can reprogram neutrophils for targeted cancer therapy.

Although research on CAR-neutrophils is still in its early phases, it benefits from several appealing features of neutrophils, such as their natural abundance, rapid migration to inflammatory sites, and various tumour-killing mechanisms, such as NETosis [191]. However, significant biological and technical challenges hinder their clinical use. Neutrophils have a very short lifespan—usually only hours to a few days—even during inflammation—and they are terminally differentiated, non-dividing cells [192–196]. These traits require frequent dosing or continuous production from human pluripotent stem cells (hPSCs) or human embryonic stem cells (hESCs) to sustain therapeutic levels, which adds to manufacturing complexity and cost [191–196]. Furthermore, the immunosuppressive TME may promote N1-to-N2 polarisation, thereby reducing the ongoing antitumor efficacy of CAR-N therapies [191].

From a genetic engineering perspective, neutrophils are known for their resistance to transduction, with transfection efficiencies often below 20%. This makes achieving stable, high-level CAR expression difficult [97, 183, 193]. Both viral and non-viral gene delivery methods can reduce cell viability or only produce transient CAR expression, thereby limiting response durability [188]. hPSC-derived CAR-neutrophils offer a renewable source for repeated or large-scale production. However, current differentiation protocols still show variability in yield and incomplete maturation, complicating standardisation and quality control [97, 193, 196]. Safety concerns include the risk of excessive inflammatory responses and cytokine release, as well as challenges in tracking neutrophil persistence and phenotype after infusion [153, 183].

CAR-N therapies show potential for targeting solid tumours but face limitations due to short lifespan, low transfection efficiency, and limited in vivo persistence [191–196]. Neutrophils only survive hours to days, leading to rapid apoptosis before they can sustain tumour engagement, which necessitates frequent infusions or continuous production from hPSC sources [97, 191–196]. Although their brief lifespan limits long-term therapeutic effects, it also offers an inherent safety feature that reduces the risk of prolonged toxicity, cytokine release syndrome, and uncontrolled growth compared with long-lived CAR-T cells [97, 193]. To improve persistence, approaches such as inserting anti-apoptotic genes (e.g., BCL-2), inflammatory pre-activation, and combining with other immune effectors (like CAR-NK cells or nanoparticle systems) are under investigation. However, sustained activation must be carefully balanced to avoid prolonged inflammation [97, 191–196].

Under GMP conditions, neutrophils cannot be expanded outside the body, requiring very high initial cell numbers, which makes hPSC-based differentiation systems preferable for scalable production [97, 193, 196]. Non-viral techniques, such as electroporation, generally yield only transient CAR expression and require large initial cell populations to achieve adequate yield. In contrast, viral methods raise concerns about integration sites and manufacturing costs [97, 193]. As a result, CAR-N cells lag behind CAR-NK and CAR-M platforms regarding scalability, persistence, and manufacturing maturity, often needing repeated infusions to maintain antitumor effects [97, 193, 196]. Still, iPSC- and hPSC-derived CAR-neutrophil platforms are being refined for standardised, off-the-shelf production, with the potential to support broader clinical applications in the future [97, 193, 196].

Recent preclinical studies show the promising potential of CAR-N strategies for solid tumours, especially GBM. hPSC-derived CLTX-CAR-neutrophils combined with TME-responsive nanoparticles have demonstrated strong antitumor effects via mechanisms including phagocytosis, ROS production, NETosis, and targeted drug delivery, resulting in significant tumour reduction and prolonged survival in mouse models [192]. CRISPR/Cas9 editing has enhanced CAR knock-in efficiency and specificity in hPSC-derived neutrophils, though limited in vivo persistence remains a challenge, leading to ongoing research to genetically prolong CAR-N lifespan while maintaining safety [192–194]. Preclinical results indicate a generally favourable safety profile, with low CRS incidence, likely due to rapid cell turnover; however, monitoring for NETosis-driven inflammation is recommended [192–194]. Combining CAR-N cells with CAR-macrophages has demonstrated synergistic antitumor effects in breast cancer models by promoting extracellular matrix degradation and immune cell infiltration, highlighting CAR-N’s role in multimodal immunotherapy [192–194].

Despite promising preclinical results, CAR-N therapies continue to face significant translational hurdles. Since neutrophils cannot be expanded outside the body, hPSC-based methods remain resource-intensive, technically challenging, and prone to batch-to-batch variability, limiting scalability and increasing production costs [193]. Additionally, low efficiency in genetic modification and heterogeneity in cell maturation make clinical standardisation difficult. Regulatory agencies must carefully assess safety risks, including off-target gene editing and inflammatory issues [191, 193]. The need for repeated doses and specialised manufacturing and infusion facilities further limits patient access. Emerging nanoparticle-based in vivo reprogramming techniques—aimed at temporarily giving endogenous neutrophils CAR-like functions—may lower costs and logistical barriers, potentially broadening access. However, their safety, durability, and effectiveness will need thorough evaluation in future clinical trials [153, 183, 191].

### Emerging CAR-engineered cell types

Beyond established platforms like CAR-T, CAR-NK, CAR-M, CAR-NKT, and CAR-N cells, a new wave of CAR-engineered immune cells is broadening the therapeutic options by utilising diverse effector mechanisms not seen in traditional methods. This section provides an overview of these emerging modalities, highlights the biological traits that set them apart from current CAR platforms, and discusses their therapeutic potential and the key hurdles to successful clinical application. These innovative cell types aim to tackle ongoing challenges in CAR-T therapy—such as antigen escape, tumour heterogeneity, poor trafficking, and immunosuppressive microenvironments—while expanding the use of engineered cell therapies for both cancerous and non-cancerous conditions.

CAR-γδ T cells leverage the innate-like, MHC-independent recognition capabilities of γδ T cells, allowing for broad tumour targeting and a lower risk of GVHD. They react quickly with potent cytotoxic responses and have shown promising activity against both hematologic and solid tumours [94, 197–200]. Recent research indicates that CAR-γδ T cells can eradicate CD19-negative leukemic clones, maintain antigen-presenting abilities, and display naïve-like memory phenotypes that support longer-lasting antitumor immunity [201, 202]. Initial clinical trials, including those targeting CD20 with CAR-γδ T cells, report encouraging outcomes in B-cell lymphomas with minimal adverse effects. Their main benefits are MHC-independent recognition and improved infiltration into solid tumours; however, obstacles such as in vivo persistence, manufacturing improvements, and large-scale clinical validation still need to be addressed [94, 197–200, 203, 204].

CAR-B cells are an innovative approach where engineered B cells act as long-lasting, antigen-specific “protein factories.” Preclinical research shows that CAR-B cells can produce tumour-targeted or bispecific antibodies, assist in antigen presentation, and enhance adaptive immune responses (203). This method is auspicious for continuous, natural antibody production without repeated doses. However, CAR-B cells are still in early development, and their long-term survival, safety, and clinical use are yet to be confirmed [198].

CAR-NKT and CAR-MAIT cells integrate swift, innate-like immune responses with adaptive killing, allowing them to modify the TME while directly attacking cancer cells. Their inherent capacity to migrate to inflamed tissues and secrete immunomodulatory cytokines makes them promising options for solid tumour immunotherapy [205]. However, their clinical application faces hurdles due to limited data, manufacturing challenges, and gaps in understanding their persistence, phenotypic stability, and potential toxicities [198, 199].

CAR-T regulatory (CAR-Treg) cells offer an immunomodulatory, not cytotoxic, function. By promoting antigen-specific immune suppression, they have the potential to treat autoimmune diseases, prevent transplant rejection, and restore immune tolerance in inflammatory conditions. Their main advantage is targeted immune regulation, but concerns include off-target suppression, risks of excessive immunosuppression, stability of the Treg phenotype, and the need for accurate antigen specificity [198, 206].

These emerging CAR-engineered cells—such as CAR-γδ T cells, CAR-B cells, CAR-NKT/MAIT cells, and CAR-Tregs—provide unique biological benefits that complement traditional CAR-T and innate immune CAR platforms. Features such as MHC-independent recognition, prolonged protein secretion, rapid effector responses, and antigen-specific immune modulation make them promising options to overcome the current limitations of CAR-T therapy. Nonetheless, advancing these platforms clinically will demand careful assessment of safety, persistence, manufacturing capacity, and effectiveness in specific contexts before they can be widely used in cancer treatments or non-malignant diseases.

### Comparative safety profiles of CAR-engineered cells

Safety is vital for the clinical success and acceptance of CAR-engineered immune cell therapies. While many CAR platforms can cause similar adverse events—such as CRS, neurotoxicity, GVHD, and on-target/off-tumour effects—their frequency and severity differ significantly based on cell type, persistence, cytokine output, and engineering strategy. This section compares the main toxicities associated with CAR-T, CAR-NK, CAR-M, CAR-NKT, and CAR-N therapies, explaining how biological features influence risk and emphasising safety benefits and challenges that influence clinical use.

CAR-T cells—both autologous and allogeneic—show the highest rates and severity of inflammatory toxicities. Severe (Grade ≥ 3) CRS occurs in 10–30% of trials [55, 56, 63–66, 207–212], with overall CRS rates ranging from 13–77% in larger cohorts [213–219]. These reactions result from substantial clonal expansion and high cytokine release after antigen engagement. Neurotoxicity (ICANS) affects up to 40% of recipients [213–219] and is more common in hematologic malignancies than in solid tumours. Well-documented on-target/off-tumour toxicities include B-cell aplasia after CD19 CAR-T therapy [41, 54, 57, 58] and rare fatal pulmonary events in early HER2-targeted CAR-T trials [58, 167]. Autologous CAR-T avoids GVHD, while allogeneic versions carry a risk that can be reduced by TCR knockout or TRAC knock-in strategies [40, 83, 178, 220–223]. Other toxicities include cytopenias, rare secondary cancers [86], viral reactivation, and tonic signalling that causes T-cell exhaustion and limits durability [8, 120, 121, 224–228].

CAR-NK cells offer a significantly improved safety profile. Rates of severe CRS stay low (0–7%) [143–147, 150, 151, 229], and neurotoxicity is rare [141, 143, 144, 146–148], thanks to controlled cytokine release and limited in vivo expansion. The lack of TCRs prevents GVHD even in the presence of HLA mismatches [142–146, 150, 230]. On-target/off-tumour effects are less common and may be mitigated by innate cytotoxic mechanisms, such as ADCC and perforin–granzyme activity [145, 147, 148, 150]. However, specific risks of the platform include uncontrolled proliferation in NK-92–based products, vector-related infectious issues, and IL-15–linked inflammatory toxicities in cytokine-armoured CARs [157, 203, 231–237].

CAR-M therapies so far show a favourable inflammatory profile, with CRS mainly limited to Grade 1–2 events and no severe CRS seen in early clinical trials [114, 134–137]. Their myeloid lineage reduces the risk of GVHD [238, 239], and on-target/off-tumour effects can be minimised by CAR designs that confine phagocytic activity to tumour-specific antigens [36, 238–240]. However, unique risks include phenotypic plasticity—such as M2-like repolarisation within the tumour microenvironment—and localised inflammatory responses linked to macrophage activation [114, 119–125, 241–243].

CAR-NKT cells exhibit low toxicity, with early trials indicating no severe CRS or neurotoxicity [168, 170, 244]. Their invariant CD1d-restricted TCR reduces the risk of GVHD [169–171], and MHC-independent recognition helps prevent off-tumour effects [37, 166, 167]. However, concerns remain about residual contamination with conventional T-cells, which could pose a GVHD risk, and the possibility of tonic CAR signalling [170, 171, 245].

CAR-N (neutrophil-based) therapies generally exhibit low overall toxicity due to neutrophils' brief lifespan and limited persistence [153, 191–196]. They do not cause GVHD [191, 238], and their innate ability to target specific cells diminishes the risk of off-tumour damage, although tissue injury in inflamed areas can still occur [153, 191, 193]. Specific toxicities include inflammation associated with NETosis and temporary increases in cytokines such as IL-6 [191–196, 243].

Overall, early clinical and preclinical data indicate that CAR-NK, CAR-M, CAR-NKT, and CAR-N platforms tend to have better safety profiles than traditional CAR-T therapies—especially regarding CRS, ICANS, and GVHD. Nonetheless, these results come from small, early-stage patient groups and need validation through larger, multi-centre trials with extended follow-up. Ongoing improvements in engineering techniques—such as TCR deletion, cytokine armouring, and universal donor systems—are vital to optimise efficacy while minimising both immediate and delayed toxicities across all CAR modalities.

## Core mechanisms of action and key challenges

CARs are modular synthetic receptors that reroute immune cells to TAAs without relying on MHC. They usually include an scFv-based antigen-binding domain, a hinge and transmembrane region, and intracellular signalling modules, often combining CD3ζ with co-stimulatory domains like CD28 or 4-1BB. This structure enables targeted activation, proliferation, and effector responses in various engineered cells, including T cells, NK cells, NKT cells, macrophages, and neutrophils. Although CAR therapies have revolutionised treatment for several cancers, their success depends on effective antigen recognition, strong and regulated signalling, and the ability to maintain cytotoxic and immunomodulatory functions in challenging tumour microenvironments. Challenges like antigen escape, poor cell trafficking, metabolic stress, and exhaustion hinder their efficacy. This section reviews the core mechanisms of CAR signalling and function, as well as the biological and microenvironmental challenges that limit the function of different CAR-engineered cell types [246–250].

### Antigen recognition and initiation of signalling

CAR–modified cells are designed to recognise TAAs via a synthetic receptor that engages antigen independently of classical MHC presentation. The extracellular single-chain variable fragment (scFv), derived from an antibody, confers antigen specificity, while the hinge and transmembrane domains transmit conformational changes upon ligand binding. Antigen engagement leads to phosphorylation of immunoreceptor tyrosine-based activation motifs (ITAMs) in the CD3ζ chain, initiating a primary activation signal that triggers downstream pathways such as ZAP-70, PI3K–AKT, and MAPK, ultimately driving transcription of effector and survival programs [251–253].

Co-stimulatory domains, mainly CD28 or 4-1BB, deliver crucial secondary signals that determine the strength, timing, metabolic health, and longevity of activation. The structural traits of these intracellular modules—such as their length, arrangement, and co-stimulatory makeup—significantly impact signalling intensity and durability. Second-generation CARs with built-in co-stimulation demonstrate increased CD3ζ phosphorylation and more controlled activation compared to first-generation versions [251–253]. Recent advances, like TCR-like CARs (TCARs), which better align with the native CD3 complex, are designed to enhance antigen sensitivity and minimise off-target effects. Although these advances exist, CAR signalling still differs fundamentally from natural T-cell receptor signalling, with distinct activation thresholds, signal integration, and a tendency toward antigen-independent tonic signalling. If not carefully tuned, tonic signalling can lead to early exhaustion and diminished effector function, highlighting the importance of designing CARs that are aligned with antigen density and the intended microenvironment.

### Cytotoxic effector functions and cytokine-mediated immunomodulation

After activation, CAR-engineered cells activate various cytotoxic mechanisms based on their immune cell platform. Lymphoid CAR products primarily utilise perforin- and granzyme-triggered apoptosis and Fas–FasL-driven extrinsic death signals. CAR-T cells are capable of serial killing, where CD8⁺ CAR-T cells mainly use granzyme B for cytotoxicity, and CD4⁺ CAR-T cells release helper cytokines. CAR-NK cells provide fast, priming-independent killing through innate cytotoxic pathways and sometimes mediate ADCC. Meanwhile, CAR-NKT cells combine perforin-based killing with chemokine secretion, such as RANTES, to attract and activate the body’s own immune effectors [254, 255].

Myeloid and granulocytic CAR platforms operate through different mechanisms. CAR-Ms primarily target tumour cells by phagocytosis and also promote adaptive immunity by presenting antigens via MHC-II and co-stimulatory molecules. Meanwhile, CAR-Ns perform additional tumour-disrupting roles by forming neutrophil extracellular traps (NETs) and releasing proteases and reactive oxygen species, which break down extracellular matrices and modify tumour structure [36, 37, 118, 144, 154, 157, 160, 191, 203, 233, 234, 244, 256–262]. In parallel with direct cytotoxicity, CAR cell products profoundly reshape the tumour microenvironment through cytokine-mediated immunomodulation. CAR-derived IFN-γ and TNF-α promote macrophage M1 polarisation, upregulate antigen presentation, and facilitate epitope spreading. Cytokines such as IL-2 and IL-15 support proliferation and the formation of memory-like or central-memory states, particularly in CAR-T and CAR-NKT cells, thereby sustaining long-term immune surveillance. Engineered “armour” strategies—which endow CAR cells with constitutive or inducible expression of cytokines such as IL-12 or IL-18—can further enhance resistance to immunosuppressive signals and improve persistence [263–265].

Nevertheless, high cytokine production is a key factor in systemic toxicities like CRS, which creates a balancing act between improving effectiveness and maintaining tolerability. To tackle this, new platforms use synthetic signalling circuits (such as AND-gate or synNotch systems) and targeted cytokine-delivery methods via extracellular vesicles or locally inducible promoters to precisely control cytokine levels. These combined cytotoxic and immune-regulating strategies turn CAR-engineered cells into versatile “living drugs” that can directly attack tumour cells and coordinate broader immune responses.

### Key challenges in the tumour microenvironment affecting CAR cell efficacy

CAR-based therapies must function within structurally complex, highly immunosuppressive TMEs. Their effectiveness is constrained not only by the inherent traits of each engineered cell type but also by external barriers such as tumour architecture, antigen variability, and persistent inhibitory signals. This section describes three interconnected challenge areas: (i) physical movement and tumour penetration, (ii) immunosuppressive and metabolic limitations within the TME, and (iii) biological challenges related to persistence and phenotypic stability across different CAR platforms [266–268].

#### Trafficking and tumour infiltration

The distribution and density of immune cells within tumours are strongly linked to patient outcomes and their response to immunotherapy [266]. For CAR-T cells, especially in solid tumours, significant challenges include inadequate trafficking and poor penetration through the extracellular matrix and abnormal blood vessels [266]. To effectively reach and spread throughout tumour tissue, CAR-T cells must navigate disorganised vasculature, high interstitial pressure, and dense stromal barriers. Approaches to improve trafficking include targeting antigens abundant in tumour blood vessels or stroma, engineering CAR-T cells to express chemokine receptors that bind tumour-derived chemokines, and combining CAR therapies with agents that modify the extracellular matrix [267].

Similar challenges impinge on innate and unconventional lymphocyte platforms. NK and NKT cells are typically found at low baseline levels and may show limited spontaneous buildup within solid tumours. For NKT cells, additional hurdles involve low cell counts, restricted infiltration, and reliance on CD1d expression by tumour or stromal cells [37, 269]. Nonetheless, as explained in Section “CAR-NKT cells”, CAR-NKT cells exhibit greater migratory capacity than conventional CAR-T cells. In a trial by Heczey et al. (NCT03294954), autologous GD2-CAR- and IL-15-expressing NKT cells (GINAKIT2) expanded in vivo, successfully targeted neuroblastoma lesions, and caused tumour regression without dose-limiting toxicities like CRS or neurotoxicity [37, 244]. Constructs with both CD28 and 4-1BB co-stimulatory domains further boosted in vivo persistence and cytotoxicity against GD2⁺ tumours and CD1d⁺ suppressive macrophages, without evidence of GVHD [259].

CAR-Ms naturally infiltrate tumour tissue and can decrease the burden of TAMs while reprogramming the remaining TAMs to adopt pro-inflammatory phenotypes. This process promotes T-cell infiltration and improves responses to other immunotherapies [36, 118]. CAR-N cells have an innate ability to migrate through peripheral tissues and extracellular matrices, allowing them to reach tumour sites quickly. They can also modulate the local environment by releasing ROS, NETs, and chemokines [191]. Overall, CAR-NK and CAR-N cells are particularly effective for rapid tumour infiltration; however, the best platform choice depends on factors such as tumour type, stromal architecture, and specific therapeutic goals [132, 144, 270–272].

#### Immunosuppressive and metabolic constraints in the TME

**Fig. 3:**
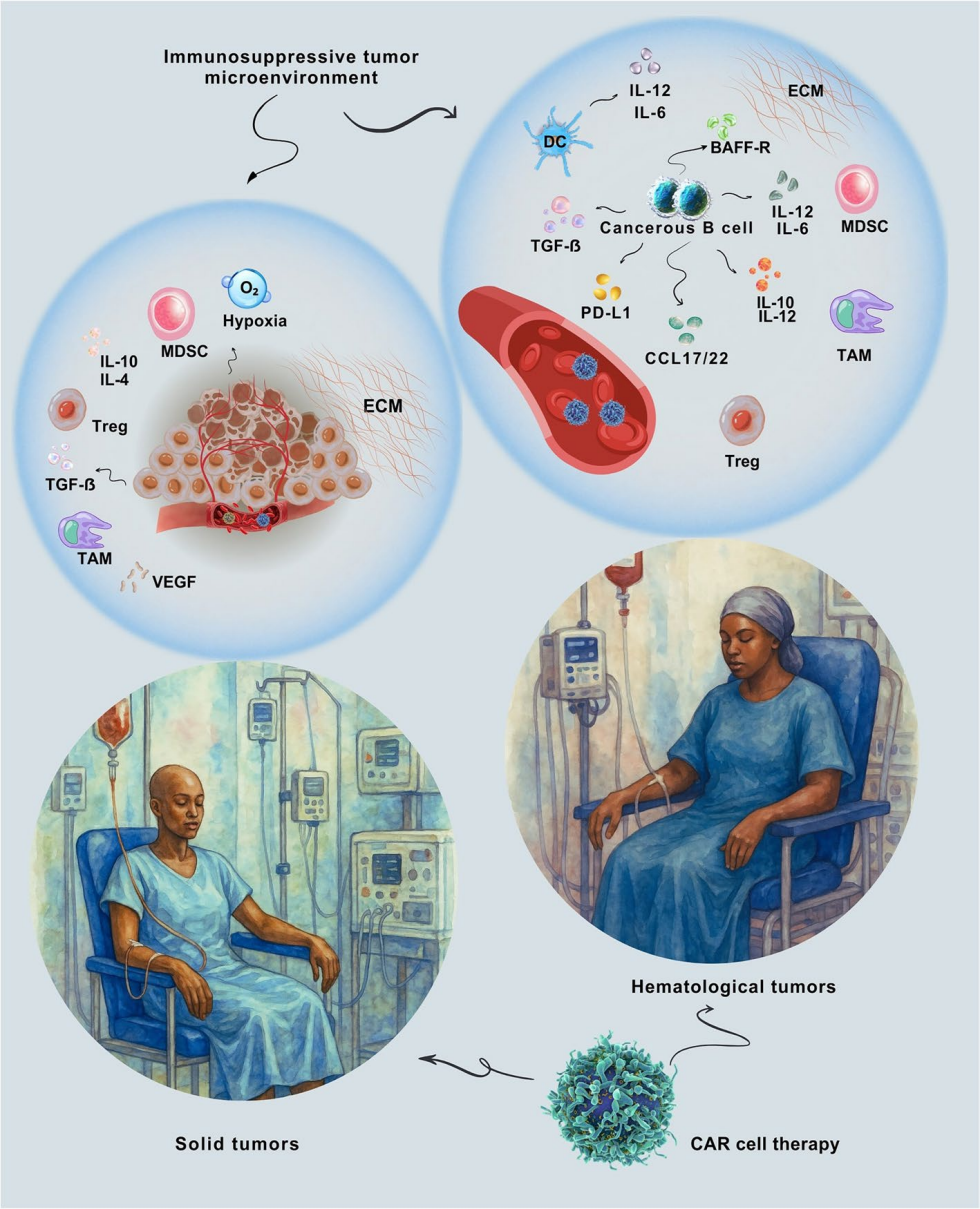
This figure shows the different immune-suppressive features of the tumour microenvironment (TME) in solid tumors (such as breast and lung cancers) versus hematological tumors (like leukemias and lymphomas). It highlights key components such as regulatory T-cells, myeloid-derived suppressor cells (MDSCs), and inhibitory cytokines that facilitate immune evasion in both types. The illustration underscores how these suppressive elements can hinder CAR cell therapy effectiveness and illustrates the specific challenges CAR T-cells face in solid tumors compared to hematological cancers. Abbreviations: MDSCs (Myeloid-Derived Suppressor Cells); CAR (Chimeric Antigen Receptor). This figure was created by the authors using Canva (www.canva.com)

The immunosuppressive TME and heterogeneity of tumour antigens present significant challenges for all CAR platforms [268]. Elevated levels of suppressive cytokines (like TGF-β and IL-10), Tregs, myeloid-derived suppressor cells (MDSCs), and TAMs, along with inhibitory checkpoint pathways, reduce CAR cell proliferation, survival, and cytotoxicity (see Fig. 3) [260]. Additionally, metabolic changes in the TME—such as hypoxia, nutrient shortages, and buildup of adenosine or lactate—further enhance immune suppression. These conditions lead to CAR-T cell dysfunctions, including exhaustion, senescence, and diminished effector functions [273]. To counter these forces, CAR-T cells are being engineered to resist exhaustion and delay senescence. This is done by modulating co-stimulatory domains, co-expressing cytokines, or disrupting negative regulators of T-cell activation [260]. Additionally, approaches that promote the formation of tertiary lymphoid structures and enhance CAR-T cell trafficking and retention within the TME are being explored [267].

NK and CAR-NK cells are similarly limited by soluble inhibitory factors and hypoxia [154, 274]. Soluble ligands for NKG2D can decrease NKG2D expression, weakening innate immune recognition. During ex vivo expansion, NK cells may also upregulate checkpoint receptors such as PD-1 or TIGIT, which can reduce their activity after transfer [274]. To address these challenges, various immune-engineering strategies have been developed, including truncated checkpoint receptors, domain-swapped activation receptors, and anti-PD-L1 CARs [154]. For instance, truncated PD-1 or PD1-based chimeric switch receptors (PD1-CSR) in NK-92 cells reprogram PD-1 signalling to send activating rather than inhibitory signals, boosting degranulation and cytokine secretion against PD-L1⁺ targets [154, 274].

The TME hinders CAR-T, CAR-NK, and CAR-M cells through various, overlapping mechanisms. CAR-T cells are particularly vulnerable to prolonged antigen exposure and tonic signalling, which increase inhibitory receptors like PD-1, TIM-3, and LAG-3; these receptors recruit SHP-1/2 phosphatases that suppress CD3ζ signalling [227, 275]. Conditions such as hypoxia and nutrient deprivation—driven by enzymes like IDO1 and arginase-1—reduce tryptophan and arginine levels, lower oxidative phosphorylation, and promote a glycolytic, exhaustion-prone state [246, 273, 275]. Moreover, TGF-β and PGE2 inhibit CAR-T activity, but resistance can be achieved by expressing dominant-negative TGF-β receptors (e.g., dnTGF-βRI) [246, 273].

CAR-NK cells are less susceptible to classic exhaustion. Still, they are significantly suppressed by TGF-β–driven downregulation of NKG2D/DAP10 through SMAD2/3 signalling, as well as by arginine depletion caused by MDSC-produced arginase-1, which reduces iNOS-dependent nitric oxide production [157, 233, 234, 276]. Additional inhibitory factors include galectin-9, soluble NKG2D ligands, PGE2, and checkpoint receptors like TIGIT, which further limit their activity [157, 203, 235–237]. Strategies such as cytokine armouring (e.g., Neo-2/15) and TGF-β receptor modification can partially restore function in these contexts [158, 234].

CAR-Ms are uniquely limited by the plasticity of macrophages. IL-4/IL-13–STAT6 signalling promotes a shift toward an M2-like, immunosuppressive state, decreasing phagocytosis and boosting arginase-1 levels, thereby increasing local nutrient depletion [121, 277]. TGF-β and PGE2 further inhibit macrophage activation and interfere with CD47–SIRPα signalling, hindering tumour cell phagocytosis [121, 157]. Unlike CAR-T and CAR-NK cells, CAR-Ms are less impacted by typical checkpoint pathways but remain highly responsive to cytokine-induced reprogramming. Strategies such as CRISPR-based stabilisation of M1-like states and IFN-γ armouring are being investigated to improve resistance against suppression within the tumour microenvironment [121, 277].

NKT cells also influence the TME. They release cytokines and chemokines, such as RANTES and MIP-1α, which attract other immune cells and can suppress immunosuppressive TAMs [257, 258, 278]. As explained in Section “CAR-NKT cells”, murine CAR-NKT cells outperform CAR-T cells in vivo by targeting CD1d-expressing M2-like macrophages, encouraging epitope spreading and activating the body's own T-cell responses. In models with high tumour burden, CAR-NKT cells may become exhausted, co-expressing PD-1 and TIM-3; blocking PD-1 has been shown to restore their activity [245]. In summary, suppression of the TME involves cell-specific mechanisms: CAR-T cells are mainly limited by checkpoint-induced exhaustion and metabolic deprivation; CAR-NK cells by TGF-β signalling and arginine metabolism; CAR-Ms by phenotypic plasticity and cytokines that reprogram macrophages; and CAR-Ns by quick N2-biased polarisation and hypoxia-related functional decline. Understanding these unique constraints is crucial for designing next-generation CARs optimised for solid TME.

#### Biological constraints on persistence and durability

Biological constraints are fundamental in determining the durability and persistence of CAR-engineered immune cells, as each platform faces inherent vulnerabilities and the suppressive effects of the TME. While CAR-T cells have the highest potential for long-term survival, their effectiveness is limited by exhaustion from chronic antigen exposure, inhibitory signals from the TME, such as TGF-β and PD-L1, and tonic signalling. In allogeneic settings, TCR-mediated alloreactivity and GVHD risks may arise, which can be mitigated by approaches like CRISPR-mediated TCR knockout or TRAC-locus insertion [178, 221]. During ex vivo expansion, telomere shortening leads to replicative senescence in both T and NK cells, limiting their lifespan post-infusion; this issue can be alleviated by TERT overexpression, though safety must be assessed [160, 279–281]. Optimising co-stimulatory domains and strategies that retain less-differentiated phenotypes also enhance CAR-T cell persistence [282–284].

CAR-NK cells typically survive only a few days to weeks, with many designs showing a median lifespan of about one week [154, 261]. This short lifespan improves safety by lowering the risk of prolonged inflammation but also restricts long-term tumour control [157, 164, 261, 285]. Their persistence is further reduced by cytokine-induced suppression and metabolic stress within the TME. Engineering strategies—such as IL-15 or Neo-2/15 armouring, dominant-negative TGF-β receptors, and gene edits that inhibit checkpoints—can enhance survival in challenging environments [157, 158, 164, 285]. However, these modifications may increase inflammation or cause off-target effects [285].

CAR-macrophages demonstrate strong local activation and effectively remodel the TME, but they lack systemic durability and have limited recirculation [285]. Their inherent plasticity allows them to switch between M1 and M2 states in response to signals like IL-4 or IL-13, which may reduce their antitumor effectiveness; efforts such as CRISPR modifications and cytokine-armoured constructs aim to stabilise M1-like activity [123, 286–289]. Conversely, CAR-N cells tend to be very short-lived, often surviving only a few hours to days due to rapid apoptosis, which might require repeated doses. Extending their lifespan using hPSC-derived sources or anti-apoptotic genes, such as BCL-2, is possible but could increase the risk of inflammation [192, 193, 290–292].

CAR-Ms demonstrate vigorous local activity and the ability to remodel the tTME, but their systemic durability and recirculation remain under study [118]. CAR-Ns tend to be short-lived naturally, which may require repeated doses and limit their long-term effectiveness against tumours [191]. CAR-NKT cells have an intermediate profile: their persistence depends on factors such as CAR design, target antigen, and TME conditions, and can be improved by IL-15 co-expression, as shown in constructs like GINAKIT2 [37, 244, 259].

In summary, CAR-T cells currently exhibit the most consistent long-term persistence but are highly prone to exhaustion and dysfunction in the TME (see Table 2). While CAR-NK, CAR-M, CAR-NKT, and CAR-N platforms provide significant safety and trafficking benefits, they generally have limited durability or face issues like plasticity and a naturally short lifespan [154, 191, 293, 294]. Addressing these biological and microenvironmental challenges with advanced gene editing, cytokine regulation, and stem-cell–based engineering will be essential for enhancing the persistence, effectiveness, and safety of future CAR-engineered cell therapies.

**Table 2:** Comparison of CAR cell therapy in solid and haematological TME

Aspect	CAR-T Therapy	CAR-NK Therapy	CAR-Macrophage	CAR-Neutrophil
Cell Source	Autologous: Patient-derived, risk of variable quality; Allogeneic: Donor-derived, risk of GvHD	Allogeneic: Derived from donors or NK cell lines like NK-92, less risk of GvHD, more consistent product	Monocytes isolated from blood can potentially be reprogrammed into macrophages	Neutrophils isolated from blood are often not genetically modified
Target Antigens	Specific TAAs (e.g., CD19, BCMA)	Lipid and glycolipid antigens, potentially multiple targets	Various TAAs, including glycoproteins and polysaccharides	Tumour cells or specific antigens (e.g., CD33)
Efficacy in Haematological Malignancies	High complete remission rates in ALL, NHL, and multiple myeloma (e.g., 80–90% in pediatric ALL)	Early studies show moderate success in AML and CLL; ongoing research is needed	Potential efficacy observed in AML and CLL, still in early research phases	Early research shows promise in targeting leukemic cells; ongoing studies are needed
Efficacy in Solid Tumours	Limited efficacy due to immune suppression; some response in relapsed/refractory settings	Promising preclinical results for melanoma and ovarian cancer	Demonstrated efficacy in preclinical models of solid tumours; ongoing clinical trials to evaluate effectiveness	Preliminary results show potential against solid tumours, including breast and lung cancer, but are still in nascent stages
Persistence	Long-lived with potential for durable responses; clonal expansion can lead to long-term remissions	Shorter lifespan; research into cytokine support (e.g., IL-15) to enhance persistence is ongoing	Moderate persistence; strategies for extending durability are under investigation, including enhanced survival signals	Short-lived (hours to days) with rapid response ability
CRS	Severe CRS due to massive T cell activation; requires careful management	Lower incidence and severity of CRS due to different activation and signalling pathways	Generally lower risk of CRS; potential local inflammatory responses are possible depending on TME	Lower risk of CRS; lower inflammation in response to specific antigens
GvHD	Significant concern with allogeneic CAR-T cells necessitates immune matching or gene editing	Reduced risk due to lack of T cell receptor (TCR) and lower likelihood of causing GvHD	Minimal risk of GvHD due to the myeloid origin of macrophages; potential for donor-derived macrophages to induce GvHD remains low	No significant risk of GvHD; predominantly myeloid origin reduces concern
Manufacturing	Time-consuming, complex, personalised manufacturing; limited scalability	Potential for ‘off-the-shelf’ use, easier manufacturing, better scalability	Manufacturing can be complex, but strategies for off-the-shelf products from differentiated iPSCs or pooled monocytes are emerging	Relatively straightforward isolation from blood, but challenges remain in maintaining function after extraction
Heterogeneity	T cells can undergo extensive differentiation, affecting potency and function	NK cells are less prone to differentiation-induced functional changes, leading to a more uniform product	Macrophages exhibit significant heterogeneity across activation states and tissue environments; controlling this heterogeneity is an ongoing challenge	Neutrophils are homogeneous but can be influenced by inflammatory signals, potentially leading to functional variability
TME Resistance	Susceptible to immunosuppressive TME; may require additional modification (e.g., PD-1 knockout)	Innate resistance to TME suppression; research into enhancing TME infiltration is ongoing (e.g., chemokine receptor modification)	Research is ongoing to enhance macrophage trafficking and function within the TME and mitigate immune suppression experienced during cancer therapy	The TME can influence neutrophils, but it also has intrinsic pro-inflammatory and anti-tumour effects; research focuses on enhancing these characteristics
On-Target, Off-Tumour Toxicity	It can be severe because the target antigens are present on healthy cells (e.g., CD19 on normal B cells)	Typically, less severe; NK cells have innate killing mechanisms that may reduce risk	May have some on-target, off-tumour toxicity due to the broad range of antigens that activated macrophages can respond to	Lower potential for off-tumour toxicity, especially in inflammatory settings, may lead to unintended tissue damage
Preconditioning	Often requires lymphodepleting chemotherapy with its own risks and side effects	May require less intensive preconditioning, lowering overall treatment toxicity	Preconditioning may not always be required, but it can enhance behaviour and functionality in specific contexts	Preconditioning may not be as essential; however, enhancing the environment for activity can be beneficial
CAR Construct	Composed of an antigen-recognition domain, hinge region, transmembrane domain, and intracellular signalling domains (e.g., CD3ζ along with CD28 and/or 4–1BB co-stimulatory domains)	Similar structure but may include NK cell-specific signalling domains (e.g., DAP10 or DAP12) to optimise NK cell activation and function	Composed of an antigen-recognition domain, it can further differentiate based on the targeted macrophage subtype	Currently, it is less common but focused on increasing the efficacy and specificity of the neutrophil response
Limitations	High potential for severe side effects; expense; limited effectiveness in solid tumors; requires advanced manufacturing	Emerging clinical data still require optimisation for solid tumour responses; longer-term effectiveness is under study	Still in experimental stages; challenges with effective targeting and potential tumour-associated immunosuppressive TME	A short lifespan limits durability; research into genetic modifications to enhance efficacy is ongoing
Unique Features	Proven success in certain haematological malignancies; ability to create a memory response	Ability to target a wide array of tumour antigens and demonstrate allogeneic applicability	Excellent phagocytic capacity and ability to mediate local inflammation in TME; can be modified to enhance efficacy	Rapid recruitment to sites of infection or tumours; can potentially utilise innate immune features for enhanced tumour targeting
Capacity for Dual Targeting	Potential for dual-targeting through co-expressing multiple CARs, enabling targeting of more than one antigen simultaneously	Capable of dual-targeting; current research focusing on enhancing specificity for different TAAs	Promising for dual-targeting by designing CARs to recognise multiple antigens, but research is still in the early stages	Possible to engineer dual-targeted CARs, particularly if neutrophils can be genetically modified, but they are still in developmental phases

Data sources: ClinicalTrials.gov for supporting trial data

## Clinical applications and efficacy in hematological and solid tumors

Although some features of the TME are common across different cancer types, CAR-based therapies face distinct challenges in haematological malignancies compared to solid tumours. In blood cancers, malignant cells are present in the peripheral blood and lymphoid organs, where vascular access is more accessible and immune effector cells can easily reach their targets. Conversely, solid tumours are surrounded by dense stromal matrices, suffer from chronic hypoxia and abnormal blood vessels, and contain immune and stromal cells that suppress immune activity, all of which hinder immune-cell infiltration and function. These structural and immune differences largely explain why CAR T-cell therapies have received rapid regulatory approval and show high response rates in blood cancers, while progress in solid tumours has been slower due to factors such as antigen heterogeneity, safety issues, immunosuppressive TMEs, and physical barriers to infiltration. Here, we review the clinical applications and effectiveness of CAR-T, CAR-NK, CAR-NKT, CAR-M, and CAR-N therapies in both blood and solid tumours, emphasising how TME influences outcomes and where new platforms could provide benefits (Fig. 3) [295, 296].

### Solid tumour treatment

In solid tumours, CAR platforms face a much more difficult environment. Factors such as dense stromal tissue, abnormal blood vessels, nutrient and oxygen deprivation, and the presence of suppressive cell populations collectively hinder the infiltration, survival, and activity of transferred cells. Consequently, the notable successes seen in blood cancers have not been achieved in most solid tumours, and current clinical research primarily centres on proof-of-concept and mechanistic studies rather than widespread application [259, 268].

#### Treatment approaches

Regulatory T cells, TAMs, and MDSCs are crucial cellular mediators that contribute to the failure of CAR-T therapy in solid tumours. They reduce cytotoxicity and enable tumour escape [297, 298]. In pediatric neuroblastoma, for instance, GD2-directed CAR-T therapy is generally safe but often does not produce lasting complete responses, even with advanced construct designs [299–302]. Trials in glioblastoma have provided proof that cytokine-armoured CAR-T cells—secreting IL-12 or IL-18—can boost local anti-tumour effects, though responses are inconsistent and often temporary [303–306]. In pediatric solid tumours, GD2-CAR-T trials reported a 63% response rate in patients with low disease burden, whereas other studies observed no objective responses despite evidence of CAR-T expansion in vivo, emphasising that tumour burden and TME composition influence outcomes [307–311]. In diffuse intrinsic pontine glioma, initial improvements were offset by severe CRS, leading to protocol changes in dosing and supportive care [312, 313].

CAR-NK therapies for solid tumours face similar challenges to those posed by the TME. Clinical trials in ovarian and breast cancers have shown remission rates usually below 25%, with responses often short-lived due to limited CAR-NK persistence and the tumour's growing resistance [154, 314]. Therefore, current efforts are focusing on combination strategies, such as radiotherapy, checkpoint inhibitors, and agents that modify the TME, to enhance the effectiveness of CAR-NK cells [314]. Several ongoing trials—like dual CAR-NK19/70 (NCT05842707, NCT05703854), TROP2-CAR-NK (NCT06066424, NCT05922930), NKG2D CAR-NK for ovarian cancer (NCT05776355), and anti-CD19 CAR-NK for SLE (KN5501)—highlight the significant preclinical and early clinical interest in using CAR-NK cells for both cancer and autoimmune diseases [315].

Early data on CAR-NKT cells in solid tumours are promising but still preliminary. These cells have demonstrated activity in preclinical models of melanoma and breast cancer, including CD1d-targeted and MUC1-directed CAR-NKTs, which reduced tumour growth and enhanced survival [37, 170, 174, 316]. As summarised in Section “CAR-NKT cells”, their capacity to combine direct cytotoxicity, modulation of the tumour microenvironment, and low GVHD risk makes them promising candidates for allogeneic therapy.

CAR-M therapies are well-suited for solid tumours because macrophages naturally infiltrate tumour sites, remodel the extracellular matrix, and coordinate local immune responses. Preclinical studies show that CAR-Ms targeting HER2 or CD20 can decrease tumour burden via phagocytosis, secretion of pro-inflammatory cytokines, and antigen presentation [36, 116, 118]. The anti-HER2 CAR-M product, CT-0508, has reached Phase I trials in HER2-positive solid tumours (NCT04660929), with early results indicating good safety and evidence of TME remodelling [37, 116, 317–319]. Additionally, CAR-N approaches, such as EGFR-targeted CAR-neutrophils, demonstrate potent cytotoxicity in lung cancer models. They can trigger local inflammatory responses, potentially enhancing their effects when combined with other treatments [37, 319].

Mechanistically, these platforms differ in how they address the hostile solid tumour environment. CAR-T cells rely on targeted cytotoxicity but are highly susceptible to antigen heterogeneity, poor infiltration, and chronic inhibition. CAR-NK cells combine CAR-mediated recognition with innate receptor signalling, which may reduce relapse driven by antigen loss, yet they still face substantial suppression by the TME. CAR-Ms operate through phagocytosis, cytokine secretion, and antigen presentation, thereby remodelling the TME and bolstering the body's T- and NK-cell responses. However, they require careful antigen selection and are influenced by macrophage plasticity. CAR-Ns enable rapid tissue infiltration and matrix disruption but are limited by their short lifespan and manufacturing difficulties [36]. Currently, CAR-T therapy remains the most advanced in clinical use, while CAR-NK and CAR-NKT show potential for toxicity reduction. CAR-M and CAR-N are emerging approaches that aim to reshape the TME and enhance the efficacy of combination therapies.

The solid TME significantly limits the effectiveness of CAR therapies compared to hematologic malignancies. Meta-analyses show that the pooled ORRs for CAR-T in glioblastoma are around 5.1%, and for solid tumours, approximately 9%, which are much lower than the often-cited 20–40% figures [320, 321]. These relatively low response rates mainly come from early-stage, single-centre trials involving carefully selected patients; factors such as prior treatments, heterogeneous antigen expression, and diverse TMEs make it challenging to interpret CAR-cell expansion and persistence. Conversely, blood cancers consistently reach ORRs of 70–90% with CD19- or CD7-targeted CAR-T cells [322–324], including an 89% ORR in CD19-positive B-cell malignancies and 87% complete remission in CD7-targeted T-cell malignancies [323, 324]. The gap is mainly due to more uniform antigen expression and easier access to the disease in blood cancers, compared with the structural barriers, antigen variability, and intense immunosuppression typical of solid tumours. Severe CRS and neurotoxicity are seen in solid tumour trials, including glioblastoma, but their frequency is strongly affected by disease site, antigen selection, and dosing strategies [321, 325].

At the mechanistic level, hypoxia in PDAC increases HIF-1α expression, which suppresses T-cell proliferation and cytokine production [326, 327]. Tumour-produced TGF-β and Treg-secreted IL-10 inhibit CAR-T and CAR-NK cell functions through SMAD and STAT3 pathways. Meanwhile, arginase-1 produced by MDSCs depletes L-arginine, an amino acid essential for sustained T-cell activation [328]. CAR-NK cells are somewhat resistant to these suppressive factors, maintaining activity in certain conditions and showing promise in preclinical models of HER2⁺ glioblastoma, although comprehensive clinical ORR data are limited [157, 329, 330]. CAR-Ms utilise phagocytosis to eliminate tumour cells and immunosuppressive cells, and to improve antigen presentation; for example, CT-0508 reduced tumour burden in ovarian cancer PDX models and may enhance CAR-T efficacy by recruiting the body's own immunity (NCT04660929) [331, 332]. However, factors like lactate buildup, fibrosis, and dense stroma hinder CAR-M infiltration and persistence in some solid tumours.

Conflicting results in GD2-CAR-T trials illustrate these complexities. One study showed a 63% ORR in patients with low-burden neuroblastoma, but no responses occurred in high-burden disease, probably due to MDSC-driven suppression and the inability of CAR-T cells to fully overcome the TME (NCT03373097) [307, 333, 334]. Response variability is also affected by factors such as antigen density and heterogeneity, patient selection, CAR design choices (e.g., CD28 versus 4-1BB co-stimulation), dosing schedules, and routes of administration. Table 3 provides a summary of clinical outcomes for CAR-T, CAR-NK, and CAR-M therapies in both hematologic and solid tumor contexts.

**Table 3:** The comparative clinical outcomes of CAR-T, CAR-NK, and CAR-M therapies

Therapy Type	Disease	ORR (%)	CRS Incidence (%; Grade ≥ 3)	Clinical Trial Number(s)
CAR-T (CD19)	ALL, NHL	80–90	70–90 (10–30)	NCT02228096, NCT02435849
CAR-T (GD2)	Neuroblastoma	20–63	10–20 (5–10)	NCT03373097
CAR-NK (CD19)	B-cell malignancies	48–73	0–7 (0)	NCT03056339
CAR-NK (HER2)	Glioblastoma	Not reported	0 (0)	NCT03383978
CAR-M	Solid tumors (preclinical)	Not reported	Not reported	Not yet registered

Data sources: ClinicalTrials.gov (e.g., NCT02228096 for CTL019 in pediatric ALL; NCT03373097 for anti-GD2 CAR-T in neuroblastoma; NCT03056339 for CAR-NK in B-cell malignancies)

As Table 3 illustrates, the high ORRs reported for CD19 CAR-T therapy in hematologic malignancies predominantly derive from early-phase trials with tightly selected patients, often with fewer prior lines of therapy, which may overestimate real-world effectiveness. Solid tumour ORRs (e.g., 20–63% in neuroblastoma) are lower and more variable, reflecting TME-mediated resistance and enrichment for patients with lower disease burden. CAR-NK therapies show moderate ORRs with minimal CRS, but available data are limited to small cohorts. CAR-M therapies remain largely preclinical. Overall, current evidence should be interpreted with caution, and larger, multicentre studies will be required to determine how these platforms perform across broader patient populations. While each CAR modality has distinct strengths and weaknesses, CAR-NK and CAR-M cells appear particularly attractive in solid tumours because of their innate adaptability to immunosuppressive environments and lower GVHD risk, whereas CAR-T retains clear advantages in hematologic disease.

#### Strategies to overcome solid TME barriers

Due to the significant barriers created by solid TME environments, recent research mainly aims to enhance CAR-cell function rather than replace platforms entirely. Armoured CAR-T cells that secrete cytokines like IL-12 or IL-18 can reprogram nearby myeloid cells to an M1-like phenotype through IFN-γ and TNF-α, which helps recruit T cells and boosts antitumour responses. For instance, Agliardi et al. [305] showed that IL-12-armoured CAR-T cells increased survival in glioblastoma models by attracting endogenous T cells and boosting local immune activation. Additionally, combination therapies combining CAR-T with immune checkpoint inhibitors, such as anti-PD-1/PD-L1, are being tested in early lung cancer clinical trials and in preclinical dual CAR-T models, although uniform improvements in ORR have yet to be observed [335, 336].

TME-modulating adjuvants, such as STING agonists, have been utilised to enhance CAR-NK function by activating innate immunity and boosting cytokine production, leading to improved tumour control in breast cancer models [337, 338]. Gene-editing techniques, such as CRISPR/Cas9 knockout of PD-1 or TGF-β receptor II, are being explored to enhance CAR-T cell persistence and to resist suppressive cytokine signalling, supported by robust preclinical evidence and early human trials in cancers such as melanoma [339–342]. However, cytokine-armed and extensively edited CAR therapies pose risks of increased systemic inflammation and off-target effects; notably, IL-12-armed products require careful monitoring for cytokine toxicity [343]. While these approaches show great potential, their optimal dosing, timing, and combination strategies are still under active clinical study.

### Addressing antigen escape in CAR therapies

Antigen escape—where target antigens are lost, downregulated, or masked—is a key mechanism of resistance to CAR therapies. It is responsible for approximately 30–50% of relapses in CAR-T–treated blood cancers and presents an even bigger challenge in solid tumours with heterogeneous antigens. Knowing how various CAR platform strategies aim to reduce antigen escape is crucial for developing effective next-generation treatments [344].

CAR-T cells, despite their notable success, remain vulnerable to antigen escape since most approved therapies target only a single surface antigen. To overcome this, dual-target and tandem CARs, along with logic-gated (AND/OR) architectures, have been created. For instance, bispecific CD19/CD22 CAR-T constructs have shown ORRs over 80% in B-ALL patients, yet relapses still happen, and antigen heterogeneity in solid tumours further restricts their effectiveness [121, 271, 344–349]. The robust memory-forming ability of CAR-T cells can maintain pressure on residual clones; however, continuous antigen exposure is necessary to preserve activity, and expanding target repertoires increases the risk of off-tumour toxicity.

CAR-NK cells combine CAR-specific recognition with innate cytotoxic mechanisms, including engagement of NKG2D and DNAM-1 ligands. This enables them to target tumour cells that lack or express low levels of the CAR-targeted antigen, providing some protection against immune escape. Their natural suitability for allogeneic, off-the-shelf use also supports widespread clinical application. However, limited persistence in vivo reduces the duration of selective pressure on tumour clones. To improve both the breadth and durability of tumour recognition, preclinical studies are evaluating dual-target CAR-NK constructs (like CD19/NKG2D) and modular antigen-sensing circuits [121, 271, 345–348, 350–352].

CAR-macrophages employ a unique approach by combining phagocytosis with antigen presentation. They can engulf tumour cells and cross-present neoantigens, which may activate the body's own T and NK cells, encourage epitope spreading, and gradually enhance antitumour responses. This approach is especially valuable in diverse tumours, where one CAR target may not identify all malignant clones. Nonetheless, keeping a pro-inflammatory, M1-like state in vivo is difficult, and shifting to an M2-like state could weaken the response or even support tumour growth. Early clinical trials are testing dual-target and logic-gated CAR-M designs, like HER2/CD47 constructs, offering a promising path toward greater specificity and sustained tumour control [121, 271, 347, 353–356].

Across various platforms, dual-targeting and advanced antigen-sensing techniques have become common solutions to combat antigen escape. CAR-T therapies remain the most clinically proven, while CAR-NK and CAR-M therapies expand the scope by leveraging innate recognition and antigen presentation, offering broader cytotoxic and immunomodulatory effects. However, none of these methods prevents antigen escape entirely, highlighting the necessity to enhance CAR persistence, expand target repertoires, and incorporate strategies that modulate the tumour microenvironment. Combining CAR-T, CAR-NK, and CAR-M therapies thoughtfully could ultimately lead to the most effective and escape-resistant tumour control.

### Synergistic combinations of CAR-cell therapies: evidence and hypotheses

The diverse strengths and weaknesses of various CAR platforms have led to increased interest in multicellular strategies that utilise multiple engineered effector populations. These approaches combine mechanisms such as phagocytosis, direct tumour cell killing, cytokine release, and antigen presentation. The goal is to address the limitations inherent to each platform and foster more sustained, comprehensive antitumor responses. A clear example of mechanistic synergy is the combination of CAR-M and CAR-T therapies. CAR-Ms not only phagocytose tumour cells but also modify the TME by secreting pro-inflammatory cytokines and expressing co-stimulatory ligands like CD80/CD86. These actions enhance the priming, infiltration, and survival of CAR-T cells. In preclinical models of HER2-positive solid tumours, combining CAR-M and CAR-T has expanded antigen recognition via epitope spreading and decreased relapse caused by antigen escape [203, 271, 357–359]. However, the longevity of these effects depends on the persistence and phenotypic stability of CAR-Ms, which has led to ongoing efforts to maintain M1-like phenotypes.

Co-administering CAR-NK and CAR-T cells offers a promising approach. CAR-NK cells deliver quick, antigen-independent killing through ADCC and innate receptors (NKG2D, DNAM-1), and may reduce CAR-T exhaustion and cytokine-release toxicity by rapidly decreasing tumour size. Preclinical xenograft studies show enhanced tumour clearance and a better safety profile when both cell types are infused together instead of separately [271, 352, 360, 361].

Additional proposed synergies involve utilising CAR-NKT cells to boost CAR-NK activity through cytokine-mediated cross-priming and epitope spreading; employing CAR-Ns to break down stromal barriers via NETosis, which helps CAR-M and CAR-T cells infiltrate more effectively; and using CAR-Treg cells to regulate inflammation, prolong CAR-T cell presence, and lessen GVHD or autoimmune toxicities in allogeneic scenarios [358, 359]. These ideas are summarised in Table 4.

**Table 4:** Synergistic CAR-Cell Combinations: Mechanistic Basis and Translational Potential

Combination	Mechanistic Rationale	Anticipated Advantages	Representative Evidence/Models	Citations
CAR-M + CAR-T	Phagocytosis, antigen presentation, TME remodelling, costimulatory ligand up-regulation	Enhanced T-cell priming and infiltration; mitigation of antigen escape	HER2⁺ solid-tumour preclinical models	[203, 271, 357–359]
CAR-NK + CAR-T	ADCC, innate receptor–mediated cytotoxicity, cytokine modulation	Complementary killing; reduced CRS and T-cell exhaustion	Xenograft combination studies	[271, 352, 360, 361]
CAR-NKT + CAR-NK	Cytokine-driven cross-priming; cooperative cytotoxic amplification	Broader antigen coverage; enhanced epitope spreading	CD8⁺ T-cell recruitment models	[358, 359]
CAR-N + CAR-M	NETosis-induced stromal disruption; phagocytosis synergy	Improved TME remodelling; facilitated infiltration and drug access	Glioblastoma and solid-tumour systems	[358, 359]
CAR-T + CAR-Treg	Immune modulation; suppression of excessive inflammation	Increased persistence; lower GVHD and toxicity risk	GVHD and autoimmune model data	[358, 359]

Preclinical studies show that such combinations can improve tumour control in specific models, especially HER2⁺ solid tumours treated with CAR-M plus CAR-T. However, these findings come from relatively uniform experimental systems that do not fully represent the clinical diversity of human cancers, previous treatments, or co-existing conditions. Applying these results clinically will require careful planning of dosing, sequencing, and cell ratios, along with strict safety monitoring. Despite these limitations, multi-platform CAR-cell strategies offer a promising approach for immune engineering. They may eventually lead to new treatments capable of producing long-lasting responses in solid tumours that have not responded to single-platform CAR therapies.

## CAR-engineered cells in non-malignant diseases

The use of CAR-engineered cell therapies is expanding beyond cancer treatment, representing a significant move toward precise immune modulation. These therapies employ synthetic receptors to adjust abnormal immune responses in various non-cancerous conditions. While initially designed to selectively destroy cancer cells with TAA, CAR platforms now also target autoreactive lymphocytes, chronically infected cells, senescent cells, and fibrogenic stromal cells, using similar mechanisms of antigen-specific killing or suppression [362–364]. Their modular design allows redirecting T cells, NK cells, macrophages, or regulatory cells toward disease-specific targets, aiming for targeted immune editing while minimising the widespread immunosuppression seen with traditional drugs. Key mechanisms involve MHC-independent recognition of surface antigens, destruction via granzyme/perforin or phagocytosis, and cytokine-fueled reprogramming of local microenvironments.

Despite these benefits, non-malignant indications present unique challenges compared to cancer: effector cells must operate in environments with lower cell proliferation, where the target load is smaller, but tissue sensitivity is greater; off-target effects on homeostatic cells or normal flora can have long-term impacts; and chronic inflammation or fibrosis can create immunosuppressive conditions similar to those in solid tumours [365]. Recent preclinical and early clinical data show that single infusions can lead to lasting remissions in some cases. Still, systematic tracking of immune recovery, infection risks, and secondary autoimmunity remains limited. Innovative approaches—such as allogeneic “off-the-shelf” CAR therapies derived from CRISPR-edited iPSCs, logic-gated constructs that activate only in response to specific antigen combinations, and armoured designs with cytokine transgenes or checkpoint decoys—are being actively developed to enhance safety, control, and access for these non-malignant uses [365].

Recent reviews highlight that CAR immunotherapies are shifting from primarily treating cancer to becoming adaptable tools for modulating the immune system in conditions like autoimmunity, infections, fibrosis, senescence, and transplantation. Early studies indicate that cellular therapies might eventually supplement or even replace long-term immunosuppressive treatments by re-establishing immune tolerance and tissue health through precise immune editing [362–365].

### Autoimmune diseases

Autoimmune diseases occur when central and peripheral tolerance fail, leading to chronic inflammation through autoantibody production, cytokine cascades, and direct tissue damage, ultimately resulting in organ dysfunction. CAR therapies, especially those targeting B-cell and plasma-cell markers, take advantage of this process by deeply depleting the harmful clones and allowing an “immune reset” with a less autoreactive immune system [18, 366, 367].

In systemic lupus erythematosus (SLE), polyclonal B-cell hyperactivity contributes to immune-complex deposition and complement activation. CD19-targeted CAR-T cells cause broad B-cell depletion, removing naïve B cells and short-lived plasmablasts while leaving some CD19⁻ long-lived plasma cells unaffected. Dual CD19/BCMA CAR-T therapies target both naïve B cells and antibody-producing cells, with 2025 Phase II results showing 95% DORIS remission, normalised anti-dsDNA levels, and sustained reductions in type I interferon signatures for over 18 months [368]. Mechanistically, B-cell aplasia disrupts feed-forward loops involving T follicular helper cells and germinal centres, allowing reconstitution with a naïve BCR repertoire characterised by predominantly IgD/IgM isotypes and reduced self-reactivity, as demonstrated by single-cell RNA sequencing [368].

Rheumatoid arthritis (RA) exhibits diverse synovial immunopathology, such as ACPA-driven recognition of citrullinated peptides and CD4⁺ responses biased towards Th1/Th17. Universal anti-FITC CAR-T cells, linked to citrullinated vimentin or fibrinogen epitopes, specifically target and destroy autoreactive B cells while sparing the rest of the humoral immune system. The 2025 EULAR Phase I results show a 70% ACR50 response rate in patients with refractory RA, along with reduced RANKL expression and osteoclast activity [368, 369]. In myasthenia gravis (MG), BCMA RNA-CAR-T (Descartes-08) removes long-lived plasma cells that produce autoantibodies against acetylcholine receptors, leading to approximately 80% improvement in activities of daily living (ADL) scores in Phase 3 trials. This treatment works by halting complement-mediated damage at the neuromuscular junction without needing lymphodepleting chemotherapy (NCT04146051, R25NS088248, NS115426-01A1).

Multiple sclerosis (MS) demonstrates the capability of CAR-T therapy to function within immune-privileged areas. KYV-101 (CD19 CAR-T) crosses the blood–brain barrier, eliminating perivascular B cells and reducing cerebrospinal fluid oligoclonal bands by about 75%. It also stops gadolinium-enhancing lesions in 85% of patients with progressive MS, according to 2025 ECTRIMS data [368, 370]. In idiopathic inflammatory myopathies (IIM) and antisynthetase syndrome, dual CD19/BCMA CAR-T approaches show promise by targeting autoantibody-producing plasmablasts and reducing IFN-γ–driven muscle fibre necrosis, resulting in normalised creatine kinase levels and partial reversal of fibrosis in recent studies [368, 371]. In systemic sclerosis (SSc), CD19 CAR-T has been linked to approximately 80% improvement in modified Rodnan skin score (mRSS) in Phase II, possibly by modulating innate immune responses, reducing NK-cell exhaustion, and lowering Scl-70 immune complexes [368, 371]. Pemphigus vulgaris, caused by B cells reactive to desmoglein-3, serves as a model for chimeric autoantibody receptor (CAAR)-T therapy: Dsg3-CAAR-T cells selectively target and destroy B cells with anti-Dsg3 BCRs through granzyme/perforin mechanisms, while sparing most of the polyclonal humoral immune repertoire [372, 373].

CAR-Tregs provide a complementary, tolerogenic aspect. FOXP3⁺ regulatory T cells engineered with tissue- or antigen-specific CARs (e.g., MOG for experimental autoimmune encephalomyelitis) can establish dominant tolerance by suppressing Th17 effector responses through IL-10/TGF-β secretion and CTLA-4–mediated modulation of APCs, thus preventing relapse in preclinical models [374–377]. Compared with biologics, CAR-based approaches offer better tissue penetration, direct cytolysis that does not depend on FcγR polymorphisms, and the potential for long-lasting effects after a single infusion through memory formation [18, 366].

Nevertheless, significant limitations still exist. To achieve sufficient CAR-cell expansion, lymphodepleting regimens are required, but their intensity must be carefully balanced against risks of genotoxicity and infections. Long-term hypogammaglobulinaemia calls for immunoglobulin replacement and close infection monitoring. Additionally, secondary autoimmunity or changes in immune repertoire may develop over time, especially in older individuals or patients with other health conditions. The development of allogeneic CAR-T therapies for SLE and RA aims to simplify manufacturing and enhance accessibility [368, 371]. Meanwhile, more precise targeting of autoreactive clones through advanced CAR designs aims to reduce off-target immunosuppression and enable sustained, drug-free remission in a broader patient population.

### Infectious diseases

Chronic infectious diseases effectively evade the immune system using antigenic variation, latent reservoirs, and regulatory pathways that suppress immune responses, allowing microbes to persist despite strong adaptive immunity [378, 379]. To counter these strategies, CAR-engineered cells are being developed to target pathogen-specific surface antigens on infected cells. This approach bypasses traditional MHC restriction and can sometimes reverse immune tolerance or exhaustion [378, 380].

In HIV infection, gp120-mediated depletion of CD4⁺ T-cells and the integration of the virus into long-lived reservoirs result in lifelong persistence despite standard antiretroviral therapy (ART). CAR-T constructs that include gp120-specific scFv domains or CD4ζ fusion receptors can penetrate lymphoid sanctuaries and eliminate latently infected cells through perforin/granzyme pathways. Phase II data show about a 60% reduction in proviral DNA (NCT04648046), though the evolution of viral quasispecies requires the development of polyspecific CARs that target multiple broadly neutralising antibodies [378, 381]. Updates for 2025 on LVgp120duo CAR-T report roughly 55% sustained viral suppression following structured ART interruption, partly due to improved NK-cell recruitment (NCT04648046).

For cytomegalovirus (CMV), glycoprotein B-targeted CAR-T products have eliminated reactivated viraemia in HSCT recipients by directly destroying infected epithelial and endothelial cells. Phase I results (NCT04227290) showed an 85% overall response rate and no GVHD, highlighting their potential advantage over antivirals for resistant strains [382, 383]. In Epstein–Barr virus (EBV) infection, CAR-T cells targeting LMP1 or gp350 can prevent or treat post-transplant lymphoproliferative disease by eliminating B-cell reservoirs. Preclinical and early clinical data (NCT00779337) indicate that about 95% of clearance occurs via FasL-mediated apoptosis [380, 384]. HBV-specific CAR-T cells targeting HBsAg have lowered viral loads in humanised mice by eliminating cccDNA-harbouring hepatocytes through IFN-γ/TNF-α antiviral pathways, with dose-finding studies ongoing [385].

Fungal pathogens are now also being considered as targets, beyond viral infections. Dectin-1 CAR-T cells that recognise Aspergillus β-glucans can induce NETosis and stimulate phagocytic responses, leading to about 85% improved survival in neutropenic models via IL-8–driven neutrophil recruitment [386–388]. Additionally, GXM-focused CAR-T cells have been shown to reduce cryptococcal CNS burden by approximately 80% in preclinical trials by exerting perforin-mediated cytotoxicity against infected cells [389, 390].

Immunologically, these strategies utilise conserved epitopes and MHC-independent recognition to target latent reservoirs, generally leading to a lower risk of severe CRS (Grade ≥ 3 < 5%) compared to oncology, since antigen burden and proliferation rates tend to be lower [335, 391–393]. However, challenges remain, including the anatomical compartmentalisation of reservoirs (such as the CNS and lymphoid follicles), antigenic variation, and the integration of CAR therapy with current antiviral or antifungal treatments. To improve persistence and broaden coverage, multispecific and armoured CAR designs (such as IL-15/IL-12 coexpression) are under investigation. Nonetheless, vigilant monitoring for superinfections and immune reconstitution inflammatory syndrome is crucial, especially in patients who are highly immunocompromised [335, 378, 380, 391–393].

### Fibrotic diseases

CAR-based immunotherapies are increasingly seen as a promising approach for fibrotic diseases, aiming to directly eliminate activated fibroblasts that drive harmful matrix buildup and organ failure. Their strength lies in targeting the core cells that cause fibrosis, overcoming the limitations of traditional antifibrotic drugs that slow but rarely reverse existing tissue scarring. Most current strategies target fibroblast activation protein (FAP), a marker expressed on pathogenic fibroblasts, enabling selective treatment with CAR-T, CAR-NK, or CAR-macrophage systems [394–397]. In animal models of cardiac, lung, and liver fibrosis, FAP-targeted CAR cells have been shown to reduce collagen deposition, lower fibroblast numbers, and improve organ function—proof that fibrosis can be actively reversed rather than just slowed [16, 394–400]. Macrophage-based therapies go further by breaking down the extracellular matrix and activating natural repair processes, shifting tissue environments toward healing rather than ongoing damage [395, 397].

A significant innovation involves using lipid nanoparticles to deliver mRNA that encodes CAR constructs directly in vivo. This method produces temporary CAR-T cells without ex vivo processing. It not only streamlines manufacturing but also minimises long-term CAR persistence, which is advantageous in fibrosis treatment, where short-term depletion of diseased fibroblasts may be enough. Extended cytotoxicity might hinder regular tissue repair. Preclinical evidence indicates that in vivo–generated CAR-T cells can effectively reverse fibrosis and restore organ function, demonstrating that a short-lived yet influential immunotherapy is feasible [26, 395, 397, 399, 401]. Additionally, CARs targeting NK cells and macrophages offer benefits such as lower initial toxicity, the potential for off-the-shelf use, and greater adaptability in dense, fibrotic tissues [395, 400].

Despite these advances, several constraints need to be considered to understand the translational landscape. Target specificity remains a primary challenge: while FAP is significantly upregulated in disease, its presence in some healthy tissues raises concerns about off-target effects. Destroying fibroblasts could also hinder vital wound healing, particularly in the cardiac and pulmonary systems [400, 402, 403]. Safety issues go beyond antigen specificity. Although cytokine release syndrome is less common in fibrotic models compared to cancer treatments, it still poses a risk, especially if CAR cells last longer than planned. Research is exploring strategies like logic-gated CARs, suicide switches, and transient mRNA expression to reduce these risks [397, 402–406]. The fibrotic environment itself creates additional hurdles. Its dense extracellular matrix, altered blood vessels, and immunosuppressive cytokines can impede CAR-cell infiltration and activity. Overcoming these challenges may require combination therapies or advanced CAR design to overcome suppression and traverse fibrotic tissue barriers [139, 271, 404, 406].

Additional challenges involve scalability and clinical application. Autologous manufacturing is still resource-intensive, and although in vivo mRNA reprogramming or NK-based systems could simplify production, practical and regulatory barriers remain [271, 397, 404, 405]. Long-term durability, immune escape, and the risk of compensatory fibrosis are not yet fully understood, as most evidence comes from preclinical studies rather than long-term clinical data [397, 400, 402, 404]. Current research aims to improve antigen selection, enhance the precision and reversibility of CAR activity, and combine these therapies with agents that influence immunity or matrix turnover. Regulatory CAR-T cells and macrophage-based constructs might not only eliminate fibrogenic cells but also help repair damaged tissues toward regeneration [398, 400]. Overall, CAR-based therapies mark a significant conceptual advance in fibrosis treatment, providing targeted, flexible, and potentially disease-modifying options where traditional therapies have limited effectiveness.

### Senescence-associated diseases

Cellular senescence is a state in which cells are permanently halted from dividing, accompanied by a secretory phenotype (SASP) that releases inflammatory cytokines, chemokines, and proteases. This process promotes “inflammaging” and contributes to age-related diseases by amplifying tissue dysfunction via paracrine signalling [407–409]. CAR-based senolytic approaches target surface markers that are upregulated during senescence, such as uPAR and NKG2DL, to specifically remove senescent cells and reduce SASP-mediated damage [14, 407, 410].

In metabolic syndrome models, uPAR-targeted CAR-T cells have been shown to eliminate p16INK4a⁺ senescent adipocytes and hepatocytes, improving insulin sensitivity with approximately 60% enhancement in glucose tolerance tests and reductions in IL-6 and TNF-α levels in aged or high-fat diet mice [14, 411, 412]. In neurodegeneration, NKG2D CAR-T cells clearing senescent astrocytes and microglia have improved cognitive performance by around 50% in Alzheimer’s disease models, partly by facilitating amyloid-β clearance and dampening neuroinflammation [407, 410]. Recent work has also shown that FAP-targeted CAR-cTregs can reverse bleomycin-induced pulmonary fibrosis in mice by eliminating pathogenic fibroblasts and remodelling the fibrotic niche with minimal CRS; however, current data do not support more detailed claims regarding SASP-specific Th2 suppression or precisely quantified functional recovery (e.g., 55%) [413].

From an immunological standpoint, senolytic CAR strategies exploit the immune-activating shift in senescent cells—marked by higher MHC I level and stress ligands—to selectively trigger cell death, with low severe CRS rates (< 5%) aligning with the relatively small proportion of senescent cells (1–15%) in aged tissues [14, 407, 414, 415]. However, the persistence of SASP factors and debris after initial clearance, along with the risk of rebound senescence, remains a concern. Armoured CARs that co-express IL-12 or other cytokines to engage phagocytes may enable more thorough removal of senescent cells and SASP components, but this must be balanced against the risk of systemic inflammation [408, 415]. Preclinical studies typically demonstrate over 70% clearance of senescent cells and functional improvements across models, and early research indicates potential for applications like osteoarthritis, where dual FAP/uPAR CAR-T cells decrease synovial senescence and joint damage [14, 408, 415]. Consequently, CAR-driven senolysis therapy targets explicitly a key factor in inflammaging, unlike gerotherapeutics, which focus on symptoms. If proven safe and effective over the long term, it could also help extend healthspan.

### Organ transplantation

In solid organ transplantation, allograft rejection results from alloreactive T-cell activation against donor MHC disparities, causing cytotoxic injury and chronic vasculopathy. Meanwhile, GVHD in hematopoietic stem cell transplantation occurs due to donor T-cell reactivity against recipient antigens [416, 417]. CAR-Tregs are being designed to re-establish tolerance through antigen-specific suppression, reducing effector responses by secreting IL-10 and TGF-β, blocking co-stimulation via CTLA-4, and generating adenosine through CD39/CD73 pathways [418].

HLA-A*02–targeted CAR-Tregs have successfully prevented skin graft rejection in humanised mouse models by suppressing CD8⁺ alloreactivity and extending graft survival by approximately threefold [416, 418, 419]. OX40L-focused CAR-Tregs, designed to target activated APCs, have reduced GVHD lethality by approximately 80% in xenogeneic models by inhibiting Th1 polarisation [416, 418, 419]. Adding 4-1BB co-stimulatory domains has enhanced FOXP3 stability even under chronic signalling conditions, resulting in roughly 90% suppression of alloresponses both in vitro and in vivo [418]. HLA-specific CAR-Tregs have consistently shown antigen-specific bystander suppression and extended allograft survival in preclinical skin and islet transplantation studies. However, comprehensive clinical data on their ability to induce liver graft tolerance, including specific success rates, are still unavailable (NCT04817774) [29, 31, 417, 418, 420–422].

The primary advantage of CAR-Treg therapies is their ability to provide donor-specific immune suppression without broadly impairing the immune system, thereby allowing continued protection against infections and cancer [417, 418]. Major challenges include Treg plasticity in inflamed tissues, where they may lose FOXP3 expression and become effector cells; to address this, strategies like chimeric cytokine receptor (CISC) engineering are being explored to stabilise IL-2 signalling and maintain their suppressive capabilities [417, 423]. Preclinical studies have demonstrated over 80% success in reducing alloresponses and significantly prolonging graft survival, suggesting that CAR-Tregs could potentially replace or complement calcineurin inhibitor therapies and support drug-free immune tolerance in some transplant cases [417–419]. Nonetheless, improving manufacturing processes, expanding capacity, and implementing long-term stability methods is essential before clinical use.

Table 5 provides an overview of representative CAR-engineered cell therapies for non-malignant diseases, highlighting promising outcomes such as high rates of drug-free remission in SLE and effective control of CMV viraemia in early-phase trials. However, these findings mainly come from small, carefully selected cohorts with few comorbidities and prior immunosuppression, which may overstate their effectiveness in broader clinical settings. Data on CAR-cell infiltration into target tissues (such as the CNS in MS or the fibrotic lung in pulmonary fibrosis) and on long-term persistence remain limited, making it difficult to assess response durability. Therefore, larger, long-term studies are necessary to evaluate safety, applicability, and the best patient groups for these innovative treatments.

**Table 5:** Summary of CAR-Engineered Cell Therapies in Non-Malignant Diseases

Disease Type	CAR Cell Type	Target	Key Outcomes	Challenges	Clinical Trial/Study
Autoimmune (SLE)	CAR-T	CD19/BCMA	Drug-free remission; B-cell reset	CRS, infections	NEJM 2025; NCT06585514
Autoimmune (RA)	Universal CAR-T	Citrullinated peptides	Autoantibody reduction	Heterogeneity	NCT06428188
Autoimmune (MS)	CAR-T	CD19	Oligoclonal band reduction	CNS access	KYV-101 Phase 1
Infectious (HIV)	CAR-T	gp120	Reservoir clearance	Escape mutants	NCT04648046
Infectious (CMV)	CAR-T	gpB	Viremia control	Latency	NCT04227290
Fibrosis (Pulmonary)	CAR-Treg	FAP/OX40L	Collagen reduction 60%	Off-target	JCI 2025
Senescence	CAR-T	uPAR/NKG2DL	Metabolic improvement	SASP	2024 models
Transplantation	CAR-Treg	HLA-A*02	Graft survival prolongation	Stability	NCT04817774; 2025

Data sources: ClinicalTrials.gov (e.g., NCT06585514 for anti-CD19 CAR-T in SLE; NCT04648046 for HIV CAR-T; KYV-101 Phase 1 for MS)

## Conclusion and future perspective

The clinical success of CD19- and BCMA-directed CAR-T therapies has catalysed a broader effort to engineer diverse immune cell types—including NK cells [269], macrophages [111], and neutrophils [183]—to overcome the limitations of T cells alone. CAR-T cells remain the most mature platform, achieving response rates of 70–90% in many haematological malignancies through targeted cytotoxicity and long-lived memory responses. However, this efficacy does not readily translate to solid tumours, where dense stroma, abnormal vasculature, antigen heterogeneity, and a profoundly immunosuppressive TME drive T-cell exhaustion, hinder infiltration, and promote antigen escape, all while increasing the risk of CRS and neurotoxicity. Clinical studies have focused mainly on conventional endpoints such as tumour response and CAR-T persistence, with relatively few trials systematically interrogating intratumoural trafficking, spatial distribution, or dynamic interactions with the TME, which remain critical but underexplored determinants of outcome [274].

CAR-NK, CAR-M, and CAR-NKT cells are emerging as promising options for addressing specific challenges of solid tumours. CAR-NK cells offer rapid, innate killing and recognise a broader spectrum of stress-induced ligands. Their lack of TCRs reduces the risk of GVHD and enables the use of off-the-shelf allogeneic products. However, CAR-NK therapies are still early in development, with issues including limited in vivo growth, short persistence within the TME, variable responses to suppressive signals, and limited long-term clinical data [36, 194]. Approaches such as selecting donors with optimal NK traits, generating cytokine-induced memory-like NK cells, and adding IL-15 or Neo-2/15 have enhanced their durability and anti-tumour effects in preclinical and early clinical trials. Nonetheless, their impact on survival and safety requires validation in larger studies [154, 194]. CAR-Ms provide a complementary approach by leveraging macrophages’ natural ability to infiltrate solid tumours, phagocytose cancer cells, and modulate the TME through cytokine and chemokine secretion [36, 111]. Early human studies indicate a promising safety profile, but further research is needed to identify optimal antigen targets, mitigate risks of M2-like polarisation, improve response durability, and develop effective combination therapies [36]. Similarly, CAR-N exhibit powerful functions—phagocytosis, reactive oxygen species production, and NETosis—but their short lifespan, difficulty in stable genetic modification, and complex production from pluripotent stem cells have slowed their progress relative to CAR-T, CAR-NK, and CAR-M therapies [183, 194, 424].

The future impact of CAR-based therapies will depend not only on next-generation engineering but also on the development of robust systems to monitor and manage these living drugs in real time. As CAR platforms diversify, effective clinical protocols will require integrated strategies to track cell persistence, tumour infiltration, phenotypic evolution, and functional status, ideally using multimodal biomarkers that combine blood-based assays, advanced imaging, and computational modelling [425, 426]. Such tools could support early detection of impending toxicities—CRS, neurotoxicity, off-target tissue damage—as well as emerging resistance or immune escape, enabling adaptive interventions such as dose modulation, switch-off systems, or adjunctive checkpoint blockade [391, 427]. In parallel, a better mechanistic understanding of how distinct CAR platforms reprogramme the TME in situ will be critical for designing rational combinations and for selecting the most appropriate effector cell type in specific tumour and non-malignant contexts.

Recent advances in CAR engineering now target not only mature effector cells but also their progenitors. Engineering haematopoietic stem cells to express CARs enables continuous production of CAR-expressing progeny, including T and NK cells, in the bone marrow and peripheral tissues. This approach helps sustain antitumor activity and addresses the limited lifespan of traditional CAR-T infusions [428, 429]. Preclinical models of CAR-HSCs demonstrate multilineage differentiation, long-term engraftment, and sustained antitumor effects, suggesting that stem cell–based therapies could provide durable immune reprogramming—provided safety issues such as insertional mutagenesis and uncontrolled clonal expansion are carefully managed [428, 429]. Concurrently, combination cellular therapies are gaining interest. For example, co-administering CAR-T and CAR-NK cells combines the antigen-targeting and memory capabilities of T cells with the rapid, MHC-independent killing of NK cells. This strategy enhances targeting of tumour clones that evade detection through MHC downregulation [430, 431]. Preclinical studies suggest that such combinations improve tumour infiltration, reduce T-cell exhaustion, and prolong treatment effects, laying the groundwork for multimodal regimens applicable to both hematologic and solid tumours [430, 431].

These innovations coincide with broader conceptual changes. CAR-engineered platforms are expanding beyond oncology to include non-malignant diseases such as autoimmune disorders, chronic infections, fibrotic conditions, senescence-related diseases, and organ transplants, where they serve as precision immunomodulators rather than solely cytotoxic agents. In these contexts, CAR-T, CAR-NK, CAR-M, and CAR-Treg therapies aim to restore immune tolerance, eliminate persistent pathogen reservoirs, reverse tissue remodelling, or promote graft acceptance, often with potential for long-lasting, drug-free remission. Early data in systemic lupus erythematosus, multiple sclerosis, HIV, and experimental fibrosis models are promising, though based on small, highly selected groups with brief follow-up, underscoring the need to study long-term safety, relapse patterns, and immune architecture in future research. From a translational viewpoint, well-structured multicenter trials comparing CAR-T, CAR-NK, CAR-M, and CAR-NKT—especially in solid tumours—are crucial to confirm hypothesised benefits in TME modulation and determine optimal applications for each approach. Simultaneously, efforts to develop multi-antigen and logic-gated constructs, in vivo CAR generation systems, and combination therapies (e.g., CAR-M with CAR-T for coordinated TME remodelling) are expected to influence future clinical strategies.

Equally important, the speed and fairness of clinical translation depend on how well the field addresses ongoing challenges such as manufacturing scalability, cost, regulatory hurdles, and global access [96, 121, 143, 147, 232, 285, 352, 432–436]. Autologous CAR-T therapies are still highly personalised and labour-intensive, taking 2–6 weeks to produce and costing over USD 400,000 per patient, with total treatment costs often exceeding USD 600,000. These timelines and costs limit access to specialised centres and usually exclude patients in resource-limited settings. While regulatory agencies rightly require rigorous, long-term safety monitoring for risks like CRS, neurotoxicity, and secondary cancers—also slowing approval and scale-up—centralised manufacturing and disjointed reimbursement systems further hinder access. On a positive note, new, decentralised, automated production methods, along with off-the-shelf allogeneic and iPSC-derived CAR products, are likely to lower costs, reduce turnaround times, and ease logistics. For example, recent initiatives in India demonstrate that CAR-T can be produced at a fraction of Western costs, showing that technological innovation and local capacity-building can significantly broaden access [96, 121, 143, 147, 232, 285, 352, 432–436].

Similar considerations apply to next-generation CAR platforms. While CAR-NK therapies are appealing due to their off-the-shelf nature and low GVHD risk, they face translational challenges, including donor variability, limited in vitro expansion (often 10–100-fold), cryopreservation-related loss of function, and high production costs ranging from USD 100,000 to 200,000 per dose. Regulatory focus is on genomic stability and biosafety, especially for gene-edited and iPSC-derived products. CAR-M therapies face additional scalability challenges because macrophages do not proliferate, requiring either extensive monocyte collections or differentiation from iPSCs, with projected costs of USD 300,000–400,000 and stringent safety assessments. CAR-NKT therapies are limited by the rarity of NKT cells (0.01–1% of circulating lymphocytes), which complicates ex vivo expansion and raises manufacturing costs to USD 200,000–300,000. UCAR-NKT strategies using engineered hematopoietic stem cells might overcome these challenges by offering off-the-shelf products, potentially cutting costs by half and reducing GVHD risk. CAR-N therapies also face hurdles: neutrophils’ short lifespan and low transduction efficiency (< 20%) often necessitate differentiation from human pluripotent stem cells, increasing costs to roughly USD 150,000–250,000 per course, with regulatory concerns about NETosis-related inflammation, transient effects, and repeated dosing. Nanoparticle-based in vivo programming and transient mRNA approaches could lower manufacturing costs by 40–50% and support single-dose treatments, but require thorough validation for biodistribution, off-target effects, and exposure control [96, 121, 143, 147, 232, 285, 352, 432–436].

Taken together, the next decade of CAR-engineered cell therapy will likely be defined by the convergence of sophisticated gene-editing technologies (e.g., CRISPR/Cas9), universal off-the-shelf platforms, multi-antigen and logic-gated designs, and rational combinations of complementary effector cell types [437, 438]. At the same time, progress will depend on harmonised international regulatory frameworks, innovative, scalable manufacturing solutions, and deliberate efforts to extend access beyond elite centres. If these scientific and infrastructural challenges can be met, CAR-based immunotherapies—including CAR-T, CAR-NK, CAR-M, CAR-NKT, and CAR-N—have the potential to redefine precision medicine across malignant and non-malignant diseases, transforming them from highly specialised interventions into broadly available, durable treatments that meaningfully improve patient survival and quality of life worldwide.

## Data Availability

Not applicable. This manuscript is a review article synthesising data from publicly available sources, such as published literature and clinical trial databases (e.g., ClinicalTrials.gov). No new datasets were generated or analysed during the preparation of this work. All referenced studies are cited in the text, and their data can be accessed through the original publications or respective repositories.
